# Admixture Increases Genetic Diversity and Adaptive Potential in Australasian Killer Whales

**DOI:** 10.1111/mec.17689

**Published:** 2025-02-28

**Authors:** Isabella M. Reeves, John A. Totterdell, Jonathan Sandoval‐Castillo, Emma L. Betty, Karen A. Stockin, Ramari Oliphant Stewart, Muriel Johnstone, Andrew D. Foote

**Affiliations:** ^1^ College of Science and Engineering Flinders University Bedford Park South Australia Australia; ^2^ Cetacean Research Centre (CETREC WA) Esperance Western Australia Australia; ^3^ Molecular Ecology Laboratory, College of Science and Engineering Flinders University Bedford Park South Australia Australia; ^4^ Cetacean Ecology Research Group, College of Sciences Massey University Auckland New Zealand; ^5^ Te Kauika Tangaroa Charitable Trust New Zealand; ^6^ Ōraka‐Aparima Rūnaka New Zealand; ^7^ Centre for Ecological and Evolutionary Synthesis, Department of Biosciences University of Oslo Oslo Norway

**Keywords:** cetacean, effective population size, heterosis, inbreeding, introgression

## Abstract

Admixture is the exchange of genetic variation between differentiated demes, resulting in ancestry within a population coalescing in multiple ancestral source populations. Low‐latitude killer whales ( *Orcinus orca* ) populations typically have higher genetic diversity than those in more densely populated, high productivity and high‐latitude regions. This has been hypothesized to be due to episodic admixture between populations with distinct genetic backgrounds. We test this hypothesis by estimating variation in local ancestry of whole genome sequences from three genetically differentiated, low‐latitude killer whale populations and comparing them to global genetic variation. We find ‘Antarctic‐like’ ancestry tracts in the genomes of southwestern Australia (SWA) population including recent (within the last 2–4 generations) admixture. Admixed individuals had, on average, shorter and fewer runs of homozygosity than unadmixed individuals and increased effective population size (*N*_e_). Thus, connectivity between demes results in the maintenance of *N*_e_ of relatively small demes at a level comparable to the sum of *N*_e_ across demes. A subset of the admixed regions was inferred to be evolving under selection in the SWA population, suggesting that this admixed variation may be contributing to the population's adaptive potential. This study provides important and rare empirical evidence that small populations can maintain genetic diversity due to sporadic admixture between different genetic backgrounds and that admixed ancestry can promote the long‐term stability of *N*_e_.

## 1 Introduction

Admixture is the exchange of genetic variation between differentiated demes and results in populations comprising ancestry from multiple sources (Korunes and Goldberg 2021). Reported examples of admixture are increasingly common in nature, and consequential changes to the genetic composition and associated demographic parameters are key to understanding the evolutionary history of admixed populations (Galaverni et al. 2017; Mathieson and Scally 2020). However, understanding evolutionary processes in admixed populations and untangling the source of their constituent ancestry components remains challenging (Tan and Atkinson 2023). Thus, despite admixture being potentially common and its evolutionary impact rapid, admixed populations remain relatively understudied (Tan and Atkinson 2023).

Admixture can shape genetic and phenotypic variation within populations (Korunes and Goldberg 2021). Potential benefits of admixed ancestry result from increased population genetic variance and can include increased fitness due to the combined ancestry of different source populations (i.e., heterosis) (Facon et al. 2005), and thereby increased adaptive potential (e.g., Verhoeven et al. 2011). For example, post‐admixture selection provides the opportunity for rapid adaptation (as reviewed in Hamid et al. 2023). In addition, admixture can mask the recessive genetic load underlying inbreeding depression (Charlesworth and Willis 2009). However, admixture can also introduce deleterious genetic variation, or mask beneficial recessive alleles within newly‐formed heterozygous genotypes leading to reduced fitness and outbreeding depression (Lynch 1991). The impact of admixture is dependent on the prior divergence, diversity, stochastic factors and ecological niche of the two admixing populations (Fontsere et al. 2019). Therefore, understanding the underlying demographic history and evolutionary outcomes of admixture is crucial within the field of molecular ecology. The first step in this process is to understand the composition of admixed ancestry within populations of interest.

Killer whales are a cosmopolitan species with a range that spans from the polar pack ice to the equator (Forney and Wade 2006). Perhaps counter‐intuitively, populations in highly productive, high‐latitude waters that support the greatest densities of killer whales (Forney and Wade 2006) are typically home to populations with the lowest genetic diversity and effective population size (*N*_e_) (Foote et al. 2021) – albeit with localized exceptions due to anthropogenic impacts on census size (e.g., Kardos et al. 2023). This global pattern is consistent with range expansion theory (Excoffier et al. 2009) and the hypothesis that these populations have colonised high‐latitude waters after the Last Glacial Maximum (Foote et al. 2021, 2019; Hoelzel et al. 2007; Morin et al. 2015). In contrast, populations with the highest genetic diversity are found at lower latitudes (Foote et al. 2021), where killer whales are typically at low density (Forney and Wade 2006; Morin et al. 2015). This has been hypothesized to be due to rare episodic admixture between divergent populations, resulting in the recombination of distinct genetic backgrounds (Foote et al. 2019). Episodic gene flow occurring amongst demes could theoretically retain *N*_e_ within each individual deme at the level of a panmictic global population (Charlesworth 2009). Even under realistic scenarios of unequal gene flow amongst natural populations, admixture has the potential to increase *N*_e_ of individual demes (e.g., Saremi et al. 2019; Kyriazis et al. 2024).

To date, population genomics studies of killer whale populations have primarily focused on high‐latitude populations (e.g., Foote et al. 2016; Garroway et al. 2024; Kardos et al. 2023; Moura et al. 2015). To better understand the processes driving the patterns in genetic diversity and *N*_e_ described above, we investigate if variation in local ancestry is consistent with admixture in genomes from three genetically differentiated low‐latitude killer whale populations (Reeves et al. 2022). Our findings shed new light on how admixed ancestry influences genetic diversity and *N*_e_ in the genomes of these populations. Furthermore, by investigating how admixed ancestry varies across the genome, we gain new insight into the evolutionary processes.

## 2 Materials and Methods

### 2.1 Sample Collection, DNA Extraction and Whole Genome Sequencing

Thirty tissue biopsies (skin and blubber) were collected under permit from 20 free‐ranging and 10 stranded animals within Australasia between 2013 and 2021. Biopsies from free‐ranging animals were taken using a Barnett crossbow with 150 lb. draw weight, 50 cm long darts (designed by F. Larsen, CETA DART, Copenhagen, Denmark) and biopsy tips (length: 40 mm), which provided samples of approximately 25 mm × 4 mm size. Tissue was preserved in 95% ethanol and stored at −80°C. The samples were previously assigned to three populations: northwestern Australia (NWA), southwestern Australia (SWA) and New Zealand (NZ) based on variation at RADseq markers (Reeves et al. 2022).

Genomic DNA was extracted using a salting‐out protocol from tissue samples (Sunnucks and Hales 1996). DNA quality, integrity and quantity were assessed using a NanoDrop 1000 spectrophotometer (Thermo Scientific), gel electrophoresis (2% agarose gels) and a fluorometer (Qubit 2.0, Life Technologies), respectively. Library build and sequencing were completed in three batches. An additional two rounds of extractions and sequencing were performed to increase the coverage of genomic data presented in Reeves et al. (2023). Firstly, DNA extracts were built into genomic libraries by Novogene (Singapore) using NEBNext Ultra II DNA library prep kit and sequenced on an Illumina NovaSeq 6000 S2 platform (150 bp PE) for a total of 547 Gb. We then built new extracts from the same samples into genomic libraries using a UDI primer set and sequenced the samples at QB3 Genomics (University of California, Berkley) across two lanes of an Illumina NovaSeq 6000 S4 platform (150 bp PE).

### 2.2 Mapping and Filtering

All bioinformatics and relevant analysis were undertaken using Flinders University's server Deepthought (Flinders University 2021). Sequences were trimmed using Adapter Removal v2.3.2 with a minimum length of 70 bp (Schubert et al. 2016) to remove trim residual adapter sequences and low‐quality stretches. Sequence data were then mapped to a high‐quality chromosomal reference genome assembly from a North Pacific ‘Biggs’ or ‘transient’ (mammal‐eating ecotype) killer whale (CNGBdb accession: CNA0050865; Kardos et al. 2023) using BWA‐MEM v 0.7.17 (Li and Durbin 2009). Duplicate and ambiguous reads were collapsed using the markdup function in SAMtools v1.14 (Li et al. 2009). Repeat regions were identified using Repeatmasker v4.1 (Smit et al. 2004), as per Foote et al. (2021) and were masked using BEDtools v2.3.0 (Quinlan 2014) to reduce potential bias to downstream population genetics inference. Separate files were generated for both autosomes and sex chromosomes for downstream analysis.

### 2.3 Published Reference Datasets

To understand the genetic relationship of the Australasian killer whales sampled and sequenced specifically for this study to other killer whale populations, we incorporated two additional datasets of published genome sequences. A dataset of 24 high coverage (> 10×) genomes, in which a single representative individual had been sequenced from populations across the species' range (Foote et al. 2021, 2023). Secondly, a dataset of low coverage (~2×) genomes, comprised of approximately 10 individuals per population from the following ecotypes: North Pacific ‘residents’, North Pacific ‘Bigg's’, Antarctic types B1, B2 and C (Foote et al. 2016). These two comparative datasets are hereafter referred to as the high‐coverage global dataset and low‐coverage ecotype dataset, respectively. Accession numbers are given in Table S2.

### 2.4 Checking for Batch Effects

To screen our data for the impact of batch effects (see Lou and Therkildsen 2022) on the different sequencing runs we estimated covariance as specified in Korneliussen et al. (2014), comparing both amongst samples and amongst sequencing runs for the same sample. Covariance was estimated using the direct sampling method implemented in ANGSD v0.935 (Korneliussen et al. 2014), in which a single allele per site is selected at random. Sites were filtered to only those that had a probability of *p* < 0.000001 of being variable, had a minimum mapping quality of 30 and a minimum base quality of 30. Sequence data for a subset of individuals from different runs were checked for consistency and batch effects (Figure S1). Sequence data from different sequencing batches for the same individual were then merged using SAMtools *merge* (Li et al. 2009) to create a single high coverage (~10×) genome per sample for use in all downstream analyses. Effective coverage (i.e., after filtering) per genome was estimated with SAMtools v1.14 (Li et al. 2009).

### 2.5 Inferring Shared Ancestry Using *D*‐Statistics

Asymmetry between Australasian populations in the sharing of derived alleles with different outgroups from the high coverage global dataset was quantified using the *D*‐statistic (Green et al. 2010). This test compares counts of shared derived alleles between an outgroup and two ingroup populations to test for statistically significant asymmetry, which would suggest either gene flow from the outgroup to one ingroup or that the assumed topology is not supported. For example, if NWA and SWA are considered ingroups and population X is the outgroup sample, the test can estimate if the data support the null hypothesis that the tree topology ((NWA, SWA,) X dolphin) is correct, and that there is no excess gene flow between X and either of the ingroups. We followed the Durand et al. (2011) definition:

$$D=\frac{\text{nBABA}-\text{nABBA}}{\left(\text{nBABA}+\text{nABBA}\right)}$$

Testing the topology ((SWA, NWA,) X dolphin), nABBA is the number of sites where an NWA genome and the outgroup X share a derived allele, whilst an SWA genome shares the ancestral allele with the bottlenose dolphin; and nBABA is the number of sites that an SWA genome and X share a derived allele, whilst an NWA genome shares the ancestral allele with the dolphin. If the null hypothesis is supported as true, we expect an approximately equal proportion of sites showing the ABBA and BABA pattern of allele sharing, and that D ≈ 0. We ran tests alternating X with a different sample from a global dataset. Statistical significance was assessed using a Z‐score based on block jack‐knife (size: 5,000,000) estimates of the standard error of the *D*‐statistics and assumes that the statistic is normally distributed with a mean of 0. *D*‐statistics were estimated in ANGSD with the following parameters and filters; ‐doCounts 1 ‐anc ‐./Tursiops.fa ‐minMapQ 30 ‐minQ 30 ‐uniqueonly 1 ‐remove_bads 1 ‐blocksize 5,000,000. These tests were implemented in ANGSD to reduce the bias of varying sequencing depth across the genome, which sampled a single base at each position of the genome using a global dataset from Foote et al. (2023); Foote et al. (2021); Foote et al. (2019). We included a bottlenose dolphin ( *Tursiops truncatus* ) as an outgroup (Short Read Archive accession code: SRX200685; (Foote et al. 2015)) which was processed as per the ‘Mapping and filtering’ section. The choice of reference genome can introduce reference bias to *D*‐statistics in the form of an artificial excess of allele sharing to the populations that are genetically closer to the reference genome. We therefore re‐estimated the *D*‐statistics using the same short‐read data mapped to an alternative high‐quality chromosomal reference genome assembly of a Norwegian killer whale (Foote et al. 2022) to check if results were influenced by the choice of reference genome.

### 2.6 Individual Assignment and Admixture Analysis

Using a pruned dataset for linkage disequilibrium (SNP ≥ 100 kb apart) we examined population structure. First, we used principal component analysis (PCA) in RStudio v2022.07.1 + 554 (Studio 2016) using package FactoMineR v2.3 (Lê et al. 2008) to identify clusters based on allele frequency covariance. We then used the maximum likelihood individual‐based assignment method implemented in NGSADMIX v2 (Skotte et al. 2013), which uses genotype likelihoods generated using ANGSD, estimating the posterior genotype likelihood based on the allele frequency as a prior, inferring the major and minor allele from genotype likelihoods and only outputting SNPs at sites that were identified as polymorphic with likelihood ratio test *p*‐values < 0.000001 and with a minimum minor allele frequency of 0.05. We removed reads with mapping quality (MAPQ) < 30, sites with low base quality scores (*q* < 20), and reads that did not map uniquely, adjusting *q*‐scores around indels, adjusting the minimum quality score to 50 for excessive mismatches and discarding bad reads (flag ≥ 256). We included available Antarctic killer whales (‘type Bs and/or ‘type C'), and North Pacific ‘resident’ and ‘Biggs' killer whale genomes to act as a reference panel (i.e., the low coverage ecotype dataset) in downstream analysis (see Table S2). We ran NGSADMIX assuming 1–8 ancestral populations (*k*) and changed the seed numbers between each of the three runs to check for convergence. We calculated Δ*k* for the number of populations using a custom R‐script.

NGSadmix along with other genetic clustering model‐based methods (e.g., ADMIXTURE and STRUCTURE) allow individual ancestries to be inferred. However, they have underlying assumptions that natural populations do not conform to, such as genetic ancestry being only comprised of populations provided; consequently, results may be misleading (Lawson et al. 2018). We therefore evaluate the fit of the admixture models based on the correlation between the true genotype and the residual difference of the genetic clustering model using evalAdmix v0.95 (Garcia‐Erill and Albrechtsen 2020). A good fit of the model results in correlation residuals close to 0. Positively correlated residuals may be indicative of similar demographic histories that are not accurately modelled (e.g., due to the inclusion of close relatives, ghost populations, incorrect K values or other demographic scenarios), whilst negative values can result from a pair of individuals that have different demographic histories but have been modelled to share the same ancestry (Garcia‐Erill and Albrechtsen 2020). Here, we used the NGSadmix outputs based on genotype likelihoods to estimate the fit of the inferred ancestry on our population dataset.

### 2.7 Inferring Shared Ancestry Using PCA

To investigate this potential admixture signal we used PCANGSD v1.10 (Meisner and Albrechtsen 2018), which can use a specified number of eigenvectors to help untangle influences of variation within the dataset. To assess the relationship between the Australasian genomes and the low‐coverage ecotype dataset, we generated a PCA inferred from genotype likelihoods in PCANGSD. Additionally, we used the PCANGSD –admix function to look at ancestry proportions of three genetically distinct low‐coverage ecotype dataset (resident, Biggs and Antarctic type C) within the three Australasian killer whales. Ancestry proportions are based on the first eigenvector (PC1) using the ‐e function based on the unpruned dataset. Genetic labelling is often imprecise and therefore misleading (Coop 2023), for instance, failing to accurately account for continuous genetic variation within a species. Therefore, we use the label ‘‐like ancestry’ (e.g., ‘Antarctic‐like’ ancestry) to infer ancestry source populations that are genetically similar to, but not necessarily genetically homogenous with, these ecotypes.

### 2.8 F‐Statistic and Treemix Tests for Ancestral Admixture

F‐statistics and TreeMix v1.12 were estimated from a VCF based on filtered BAMs to generate a VCF containing called SNPs using BCFtoolsv1.9 based on the low coverage dataset and Australasian genomes (Danecek et al. 2016). SNPs were kept if they were called in at least 80% of individuals, had an allele balance between 20% and 80% within the heterozygote genotype, and exhibited a minor allele frequency of at least 3%.

The *f*_3_‐statistic uses variation in allele frequencies of population pairs within a tree to assess genetic drift (Patterson et al. 2012; Peter 2016; Reich et al. 2009). The *f*_3_‐statistic can detect admixture, even if gene flow occurred several hundred generations ago (Patterson et al. 2012). These tests are estimated based on *f*_3_(A; B, C), where a statistically significant negative *f*_3_‐statistic indicates admixture in population A. *F*_3_‐statistics were estimated as per Patterson et al. (2012) with standard errors using jackknife blocks of 1000 SNPs using TreeMix. Foote et al. (2021) estimated a mean inverse SNP density (Kb per SNP) of 0.642 (range: 0.204–4.906) from the high‐coverage dataset of killer whales. Thus, physical block size is expected to fall within the range of 0.2–5.0 Mbp. We tested three *f*_3_‐statistics forms for SWA, NWA and type C, rotating the position in the form of *f*_3_(A; B, C).

When admixture has been detected, the admixed proportions can be estimated using *f*_4_ ratio statistics. The tests based on the form *f*_4_(A, B; C, D) are expected to be zero when there is no overlap in the paths of allele frequency changes between A and B and between C and D in the tree edges. *F*_4_ indicates admixture when the value statistically significantly deviates from 0, negative or positive estimates are dependent on the position of the source population on the tree. This statistic is insensitive to post‐admixture drift, making it useful for detecting admixture events, even if they occurred hundreds of generations ago (Patterson et al. 2012).

Patterson et al. (2012) defined the *f*_4_ ratio as:

$$\;f4\frac{\left(C,O;X,B\right)}{\left(C,O:A,B\right)}$$

where A and C are a sister group, B is sister to (A, C), X is a mixture of A and B and O is the outgroup. This ratio estimates the ancestry from A, denoted as α, and the ancestry from B, as 1–α identify source populations of admixture with the three Australasian populations we tested *f*_4_, as per Patterson et al. (2012) with standard errors using jack‐knife blocks of 1000 SNPs. We tested three *f*_4_‐statistics in TreeMix where the Australasian populations and Antarctic type C were rotated in the form of *f*_4_(A, B; C, D).

Genetic relationships amongst the reference and Australasian populations were reconstructed using population allele frequency data to generate a bifurcating maximum likelihood tree to illustrate genetic drift using TreeMix v1.12 with Gaussian approximation (Pickrell and Pritchard 2012). The tree's branches represent population relationships based on predominant alleles. Migration edges are introduced between populations that deviate from the tree model, indicating inferred allele exchange. The inferred directionality of gene flow along migration edges is based on asymmetries in a covariance matrix of allele frequencies relative to an ancestral population, as indicated by the tree. We used TreeMix to generate a bifurcating maximum likelihood tree using SNP blocks of 1000 SNPs based on a VCF file for migration events 0–2 to understand potential directional admixture events.

### 2.9 Local Ancestry Inferred From Haplotypes

We used ELAI v1.01 based on a two‐layer hidden Markov Model to detect the structure of haplotypes based on linkage disequilibrium to infer the local ancestry of admixed individuals based on unphased data (Guan 2014) from the VCF file. The program performs independent phasing before calling local ancestry (Guan 2014). Thus, we leveraged this to understand the SNP dosage from the source populations within the admixed populations, using a three‐way admixture model from three source populations with ‐C (upper clusters) 3 ‐c (lower clusters) 15 based on 5 × C as per ELAI documentation and mixed generations (mg) of 10 and 100 for comparsion. Source populations ideally need to be highly differentiated from one another, and therefore we choose population reference datasets that represent resident‐like, Biggs‐like and Antarctic‐like ancestry (type C). We used a custom R script to visualise the dosage along the genome with 50 kb sliding windows and 10 kb stepping size.

### 2.10 Tests for Recent Admixture and Timing

Approaches that model the global proportion of an individual's genome based on hybrid indexes or admixture proportions cannot disentangle recent (i.e., within the last five generations) and ancestral admixture (Lawson et al. 2018). The ‘Admixture Pedigrees of Hybrids’ (apoh) approach (Garcia‐Erill et al. 2023) assesses inter‐ancestry heterozygosity to detect recently admixed individuals and estimate possible scenarios of recent admixture. This approach uses two complementary models. The paired ancestries model analyses unordered paired ancestries without distinguishing between the possible heterozygous permutations (as reviewed in Garcia‐Erill et al. 2023). Whilst the parental admixture model assesses the ordered paired ancestry and considers the possible permutations, apoh automates these models and considers the relative fit of the estimated proportions to detect recent admixture (as reviewed in Garcia‐Erill et al. 2023). Therefore, we estimated recent admixture with the possible source populations. Note, as we were trying to understand recent admixture events, we included all population‐level whole genome data, including the three Antarctic contemporary populations in this analysis, therefore using K = 7 NGSadmix input.

### 2.11 Calling Runs of Homozygosity for Individual Genomes

Runs of homozygosity (ROH) were identified using a window‐based approach in plink v1.07 (Purcell et al. 2007) for the Australasian populations on a VCF file. We used the high‐coverage global dataset when calling the ROHs to act as a reference dataset. Using a sliding window size of 300 kb, a minimum of 50 SNPs with a density of one SNP per 50 kb was required to identify a ROH in the dataset. Up to three heterozygote sites per 300 kb window within called ROHs were allowed to account for genotyping errors, and a 1 kb distance between two SNPs was necessary for them to be considered in separate ROHs.

### 2.12 Tests for Selection for Admixed Loci

In an attempt to assess if any regions that were admixed were under selection, we used PCANGSD v1.10‐selection function that extended the model of FastPCA that uses genotype likelihoods of the Australasian and low coverage ecotype dataset (Galinsky et al. 2016) to perform a genome‐wide selection scan along PC1 of the unpruned data (Meisner et al. 2021). We fit the data to a qqplot in Rstudio (RCoreTeam 2022), using chi‐square and Lamba to explain variance that statistically significantly differentiates from genetic drift along the axis. Additionally, we examined any peaks driven by the variance in PC1 using package qqman (Turner 2014) to build a Manhattan plot with the −log10 transformed *p*‐values obtained and plotted against chromosome and position. The data were not pruned to remove the effects of linkage disequilibrium (Lotterhos 2019). Therefore, it is important to consider the variance driven by each PCA is inflated by the inclusion of non‐independent SNPs.

## 3 Results

### 3.1 Genome Datasets and Batch Effects

To understand how different library build methods, sequencing platforms and sequencing service providers may bias results between and within runs, we compared replicates between runs estimated with 783,990 SNPs based on genotype likelihoods. Figure S1 illustrates the covariance of replicates within and between runs, as well as replicate samples, sequenced on different platforms, clustered closely together in the adjacent tree. Thus, whilst genotyping may exhibit some small variability between runs, batch effects are unlikely to influence the biological significance of any results here.

### 3.2 *D*‐Statistics Indicate Admixture Signal for Southwestern Australian Killer Whales

**FIGURE 1:**
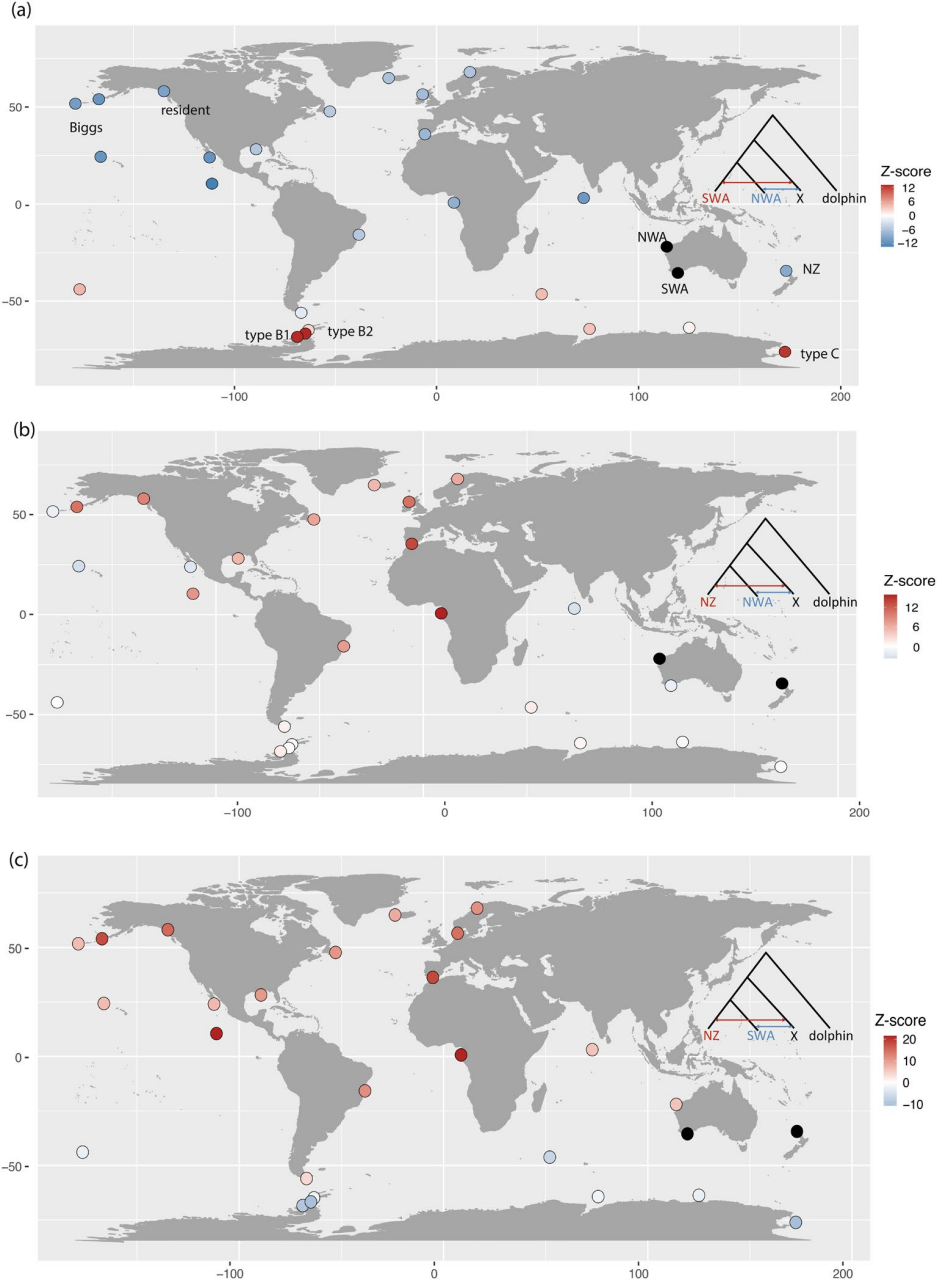
A comparison of *D*‐statistic form **(a)** *D*(SWA, NWA, X, dolphin), **(b)** *D*(NZ, NWA, X dolphin) and **(c)** *D*(NZ, SWA, X dolphin) for Australasian killer whales compared to a global reference dataset. Statistical significance is indicated by a Z‐score > 3 or < −3. ‘SWA’ is southwestern Australia, ‘NWA’ is northwestern Australia and ‘NZ’ is New Zealand. Dots are representative of the sample locality.

We found that 21 of 25 tests of form *D*(SWA, NWA; X, dolphin) were statistically significant based on the Z‐score of −3 < Z > 3 and that in 16 of these tests, X (representing a geographically widespread set of populations, Figure 1a, S2a) shared a statistically significant excess of derived alleles with NWA. The highest *D*‐statistics being when X was one of the distinctive Antarctic morphotypes (types B1, B2 and C) (Figure 1a, S2a).

Additionally, we found 11 of 25 tests of the form *D*(NZ, NWA, X, dolphin) were statistically significant due to an excess of shared derived alleles between the NZ genome and genomes from the North Atlantic, North Pacific resident, offshore, Clipperton Island populations (Figures 1b and S2b). The NWA genome shared an excess of derived alleles with genomes from the Maldives, Hawaii and Mexico (Figures 1b and S2b). These *D*‐statistic results mirror the genetic affinities of each Australasian genome in the PCA in Figure 1c of Foote et al. (2019). Re‐estimation of the *D*‐statistics after re‐mapping to an alternative reference assembly generated from a Norwegian killer whale (Foote et al. 2022) provided comparable results (Figure S3, Tables S4–S6), indicating no major impact of reference bias on our findings.

Estimation of *D*(NZ, SWA, X, dolphin) revealed that 21 of 25 of the tests statistically significantly deviated from the expected symmetry of derived allele sharing under the tested topology. The same populations that shared an excess of derived alleles with the NWA genome relative to the SWA genome also shared an excess of derived alleles with the NZ genome (Figures 1c and S2b). Likewise, the SWA genome also shared an excess of derived alleles with the Antarctic types in this test. Thus, we see that the NWA and NZ genomes share a genetic affinity with populations to the North, whilst the SWA genome shares a genetic affinity with populations to the south.

To test whether X should be an ingroup with an Australasian population we estimated *D*‐statistics of the form (pop1, X; pop2, dolphin), where pop1 and pop2 are Australasian populations (Figure S4). Consistently, the highest significance was for tests including an Antarctic type as an ingroup (X). Australasian killer whale genomes share a statistically significant excess of shared derived alleles relative to sharing between Australasian and Antarctic genomes. Additionally, like the previous *D*‐statistic tests, genomes from the NZ and NWA populations share a statistically significant excess of derived alleles with various northern hemisphere populations, whilst the SWA population share an excess of derived alleles with a single Southern Ocean population (See Table S6 and Figure S4 for population‐specific results).

In summary, based on the above results from all *D*‐statistic tests, there is statistically greater Antarctic‐like ancestry in the SWA population relative to the NZ or NWA populations. However, the three Australasian populations share more ancestry with each other than any does with the Antarctic populations.

### 3.3 Population Assignment

**FIGURE 2:**
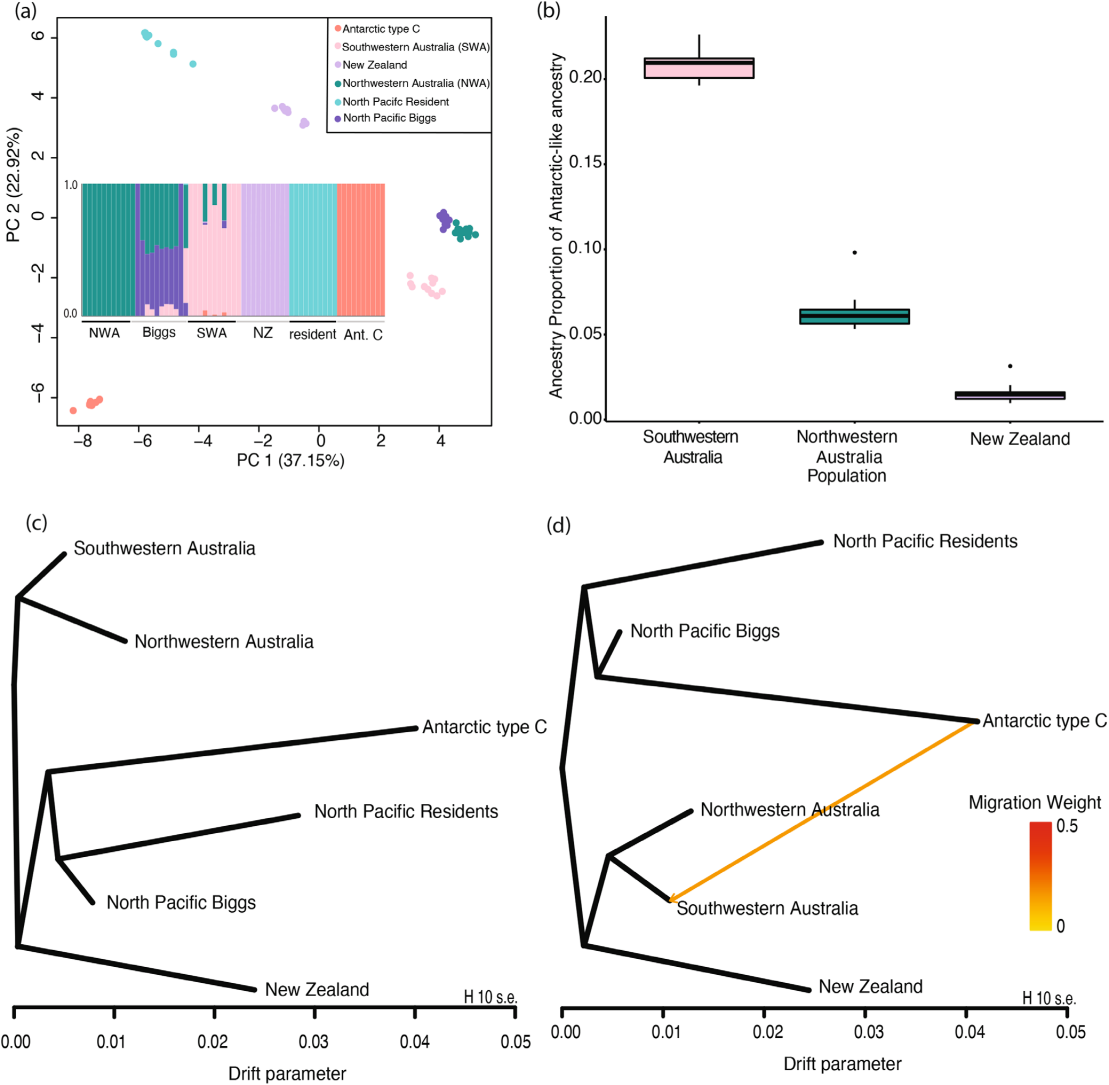
**(a)** Principal components analysis and NGSadmix plot (K = 6) of Australasian killer whale genomes and reference population‐level data available from Foote et al. (2016) based upon genotype likelihoods from 18,355 pruned SNPs. Variation along PC1 was used to quantify the **(b)** ancestry proportion of Antarctic‐like ancestry within Australasian populations using genotype likelihoods SNPs. **(c)** TreeMix maximum likelihood graph from whole‐genome sequencing data (1,093,713 unpruned VCF‐called SNPs). Horizontal branch lengths are proportional to the amount of genetic drift that has occurred along that branch. The scale bar shows 10 times the average standard error of the entries in the sample covariance matrix. **(d)** TreeMix maximum likelihood graph with directional migration edge inferred using TreeMix and coloured by migration weight.

Population assignment was estimated from genotype likelihoods from 18,355 unlinked SNPs. Congruent with Foote et al. (2019), results from Figure 2a (see Figure S5 for additional type B genomes) demonstrate that the greatest differentiated clusters are the Antarctic killer whales along PC1 (37.15% explained variance), with the residents driving the variance along PC2 (22.92% explained variance). This pattern is also consistent when assessing the additional type B1 and B2 genomes, however, variation is increased along PC1 (56.15%) and decreased along PC2 (14.75%). We used PCANGSD to highlight that SWA shares a higher proportion of Antarctic‐like ancestry (one‐way ANOVA, *p* < 0.05) relative to NWA and NZ (Figure 2b) based on the variation explained on PC1.

NGSadmix reflected the same hierarchical structure as seen in Foote et al. (2019) with K = 2 and K = 3 being the best‐supported number of genetic clusters (Figures S6 and S7). EvalAdmix highlighted residual differences within populations inferred by NGSadmix, suggesting that the data violate at least some of the assumptions of the underlying model (Figure S8). These assumptions encompass Hardy–Weinberg equilibrium, random mating, linkage equilibrium, the comprehensive inclusion of all potential source populations, unrelated individuals and the independence of alleles at loci (Ainley and Blight 2009; Garcia‐Erill and Albrechtsen 2020). EvalAdmix correlates residuals between true and predicted genotypes to identify violations of the admixture type models. Here, some samples from the Australasian and resident populations have positive residual differences within their population, suggesting high relatedness which was not unexpected based on their small population sizes. Positive residuals may also reflect unrepresented ghost populations. However, three residents (#ID 8–10), from the southern resident cluster have negative residual differences with the rest of the population, likely due to shared population‐specific demographic histories, which include inbreeding (see Kardos et al. 2023). Similarly, a single Antarctic type C individual (#ID 3) appears to have negative residuals with the individuals in the cluster and appears to have the lowest coverage genome from the population. The remainder of the samples demonstrate close to zero if not zero residual differences, suggesting that samples generally are a good fit to the NGSadmix model.

### 3.4 Evidence for Ancient Admixture Between Distinct Genetic Backgrounds

The statistical test of the form *f*_3_(SWA; typeC, NWA) was negative suggesting admixture, but was not statistically significant (*Z*‐score of −2.06; Table S9). *F*_4_‐statistics highlighted the complexity of relationships, for example, with *f*_4_(SWA, typeC; NWA; NZ) showing significance (*Z*‐score of −17.75; Table S10). However, these tests were less suitable for hypothesis testing in this case than the *D*‐statistics.

TreeMix graphs reflect that resident, Biggs and Australasian populations cluster together consistently across all tested trees (Figures 1d,e and S9). However, the position of New Zealand and Antarctic populations in the topology changes depending on the number of migration edges. The migration edges support the *D*‐statistics, with inferred gene flow of Antarctic‐like ancestry into SWA and resident‐like ancestry into the NZ population. However, it is important to note the residual fit of the observed data versus the predicted squared allele frequency difference demonstrates that none of the topologies perfectly fit the data (Figure S10). TreeMix models are complex, and outputs are for a single best‐fit model when there may be several alternative evolutionary histories that fit the data almost as well but which are not outputted by the program. However, the TreeMix results are broadly consistent with the suite of alternative and less parameterized analyses run here. Specifically, the *D‐*statistics, TreeMix and NGSadmix provide evidence that there is a clear and consistent signal of Antarctic‐like ancestry in the SWA genomes arising from admixture.

**FIGURE 3:**
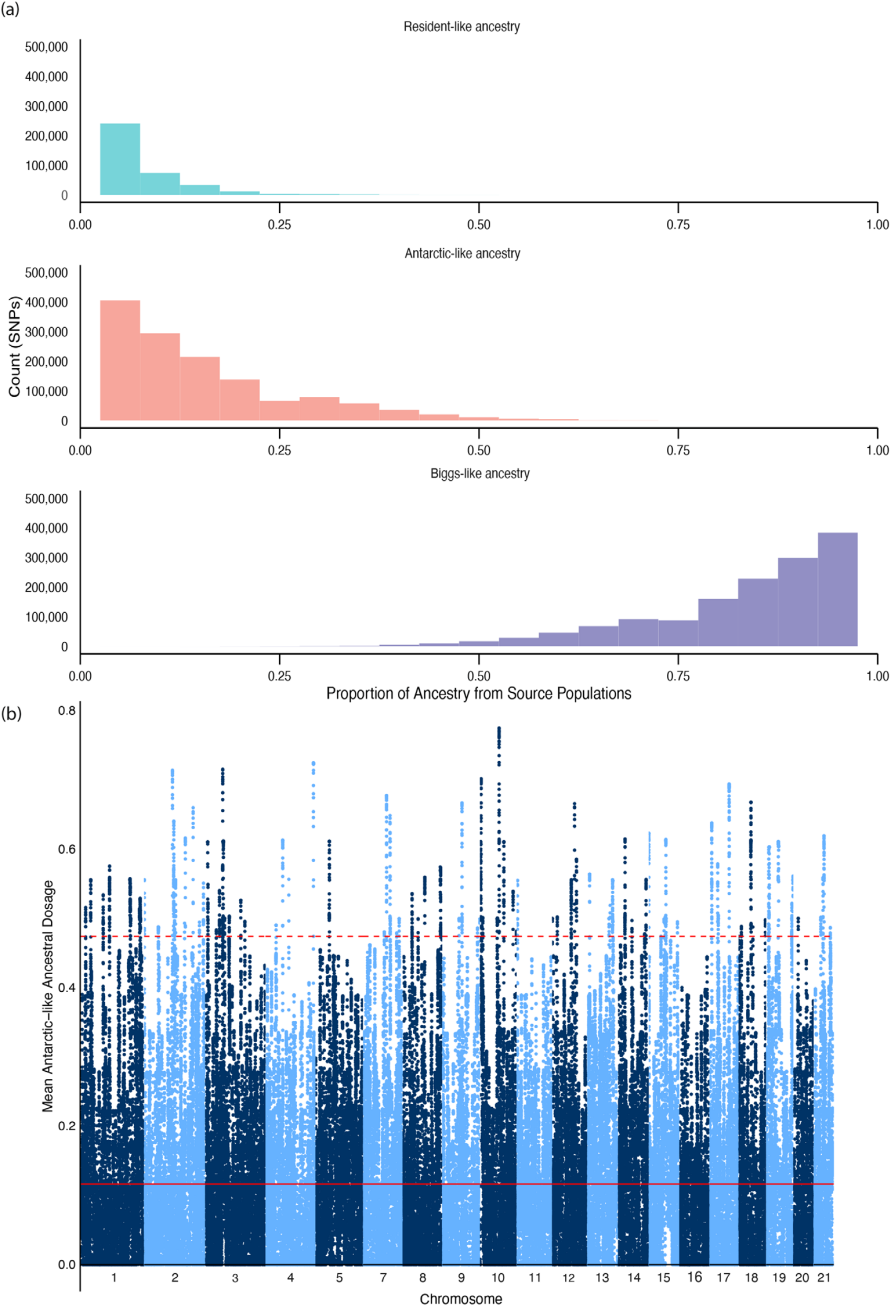
The distribution of local ancestry in the southwestern Australian population by SNP (*n* = 1000,517) by **(a)** each of the three reference panel populations for SNP dosage genome‐wide and **(b)** mean Antarctic‐like ancestry across the genome per chromosome for 100 mixing generations. The solid red line is the mean ancestry proportion, and the dotted lines are ±3 standard deviations based on 50 kb sliding windows and a step size of 10 kb.

To examine the extent of ancient admixture (*n* generations 100 and 10) we used ELAI to infer local ancestry and its dosage from the reference population into recipient populations. ‘Biggs‐like’ ancestry was the major ancestry component within the SWA population, suggesting a recent common ancestor between the SWA and North Pacific Biggs killer populations (Figures 3a and S11). The highest minor ancestry component within the SWA genomes was represented by the Antarctic‐like ancestral dosage. The distribution of Antarctic‐like ancestry found at high frequencies in the SWA population is in small blocks along the genome, which suggests it is not recent in origin (Figure 3b).

### 3.5 Evidence for Ongoing or Recent Admixture

**FIGURE 4:**
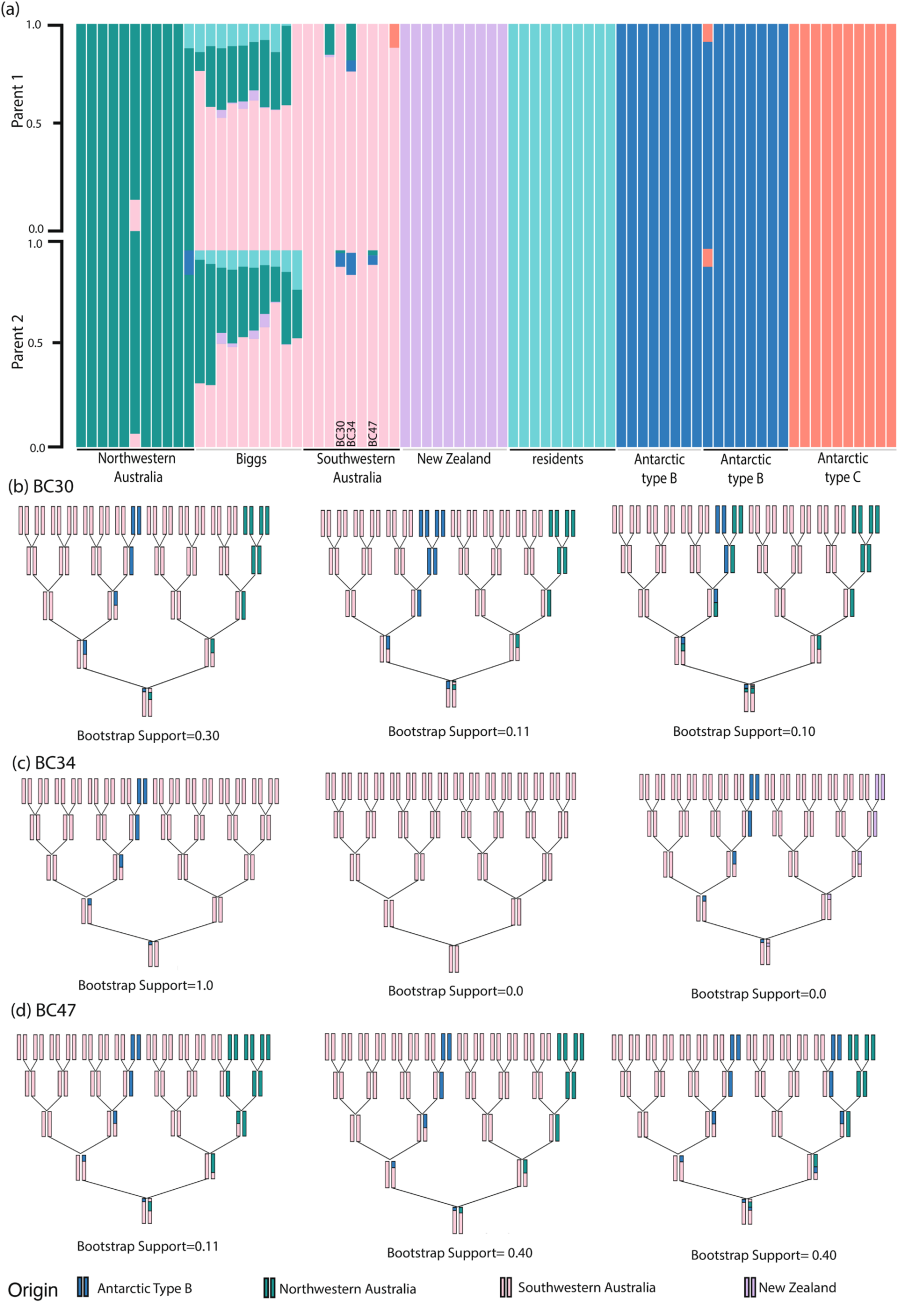
Estimates for recent admixture of three candidate admixed southwestern Australia killer whales assuming K = 7 ancestral components. **(a)** Admixture proportions using the parental admixture model in apoh using only the offspring's genotypes for the candidate and **(b)** three most compatible recent admixture pedigrees, including their bootstrap support based on 18,355 SNPs with bootstrapping.

To verify if any admixture was recent (i.e., within the last five generations), we focused on three candidate samples (BC30, BC34 and BC47 from southwestern Australia) that through population assignment tests appeared to have ancestry arising from recent admixture (see Figures 2a and S12). These candidate samples' parent admixture demonstrated that all parents were estimated to have consistent admixture patterns, with clear remnants of ‘Antarctic‐like’ ancestry (Figure 4). When modelling recent admixture within the last five generations for the candidates we used the three best‐fit pedigrees and contrasted them with three not recently admixed samples (BC14, BC28 and BC51) (Table S11, Figure S13). Whilst comparing the three selected candidates that were suggested to not be recently admixed, we find that the inconsistency index and distance to independent pedigrees are marginally higher (Table S8), supporting the lack of recent admixture within these three samples (BC14, BC28, BC51; Figure S13). Figure S14 demonstrates the potential admixture of a single NWA animal; however, it does not support admixture with any of our low‐coverage ecotype population data. The admixture index infers that outbreeding occurred between 2.95 and 4.19 generations ago (≤ 100 years based on a generation time of 25.7 years, Taylor et al. (2007)). Therefore, it appears that at least some SWA animals carry ancestry inherited from a great grandparent or great great grandparent with Antarctic‐like ancestry.

### 3.6 Admixture Affects Genetic Diversity

**FIGURE 5:**
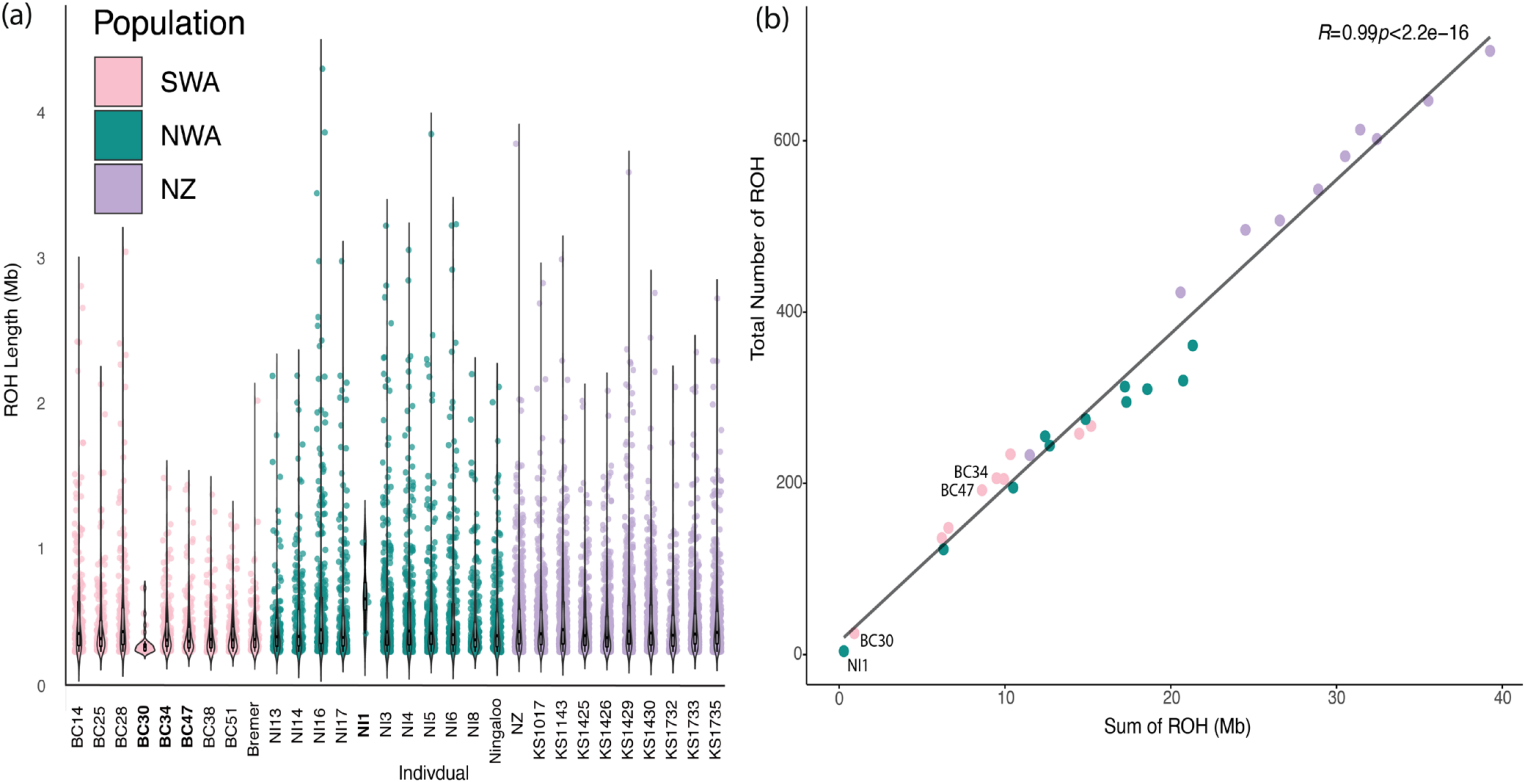
Genetic diversity estimates of Australasian killer whales. (a) Kernel density (violin) plots of the length of individual ROH in each genome based on 1000,517 SNPs. White rectangle shows the interquartile range, and the black bar is the median of the data and (b) depicts the number of ROH compared to the sum of the length of ROH across the autosomes ‘SWA’ is southwestern Australia, ‘NWA’ is northwestern Australia and ‘NZ’ New Zealand. The admixed individuals are labelled in bold font, in (a) and labelled in (b). Note the ‘NZ’, ‘Ningaloo’ and ‘Bremer’ labelled samples are from Foote et al. (2021).

ROHs were estimated to understand how recent admixture may be influencing genetic diversity and *N*_*e*_ of populations. Admixture can break up haplotypes that form ROH, thereby reducing ROH in the genome (Ceballos et al. 2018) and increasing heterozygosity (Charlesworth 2009). Statistically significant differences in ROH lengths were observed amongst Australasian populations (mean ROH length in kb; NWA: 565 ± 25.64SD, NZ: 536 ± 12.43SD, SWA: 489 ± 20.46SD; one‐way ANOVA, *p* < 0.05, see Figure 5a) based on 1000,517 SNPs. We directly compare the three samples that appeared recently admixed (Figure 2a, BC30, BC34 and BC47), with three seemingly unadmixed individuals (BC14, BC28 and BC51) from the population. The mean ROH for recently admixed individuals was 425 kb, compared with 524 kb for other SWA individuals (one‐way ANOVA, *p* < 0.05; Figure 5a).

The sum and number of ROH (SROH and NROH, respectively) within individual genomes were highly correlated (R = 0.99, *p* = < 2.2–16; Figure 5b). SROH and NROH reflect population demography (Ceballos et al. 2018). Larger populations tend to have lower SROH and NROH, which are further reduced by admixture, particularly between divergent lineages with different haplotypes (Ceballos et al. 2018). In contrast, populations experiencing bottlenecks show shorter and fewer ROHs, as seen in NZ (Foote et al. 2021). As expected, the more admixed the individual, the less the number and sum of ROHs, as evident in BC30 and NI1.

### 3.7 Potential Signal of Selection of Admixed Regions

**FIGURE 6:**
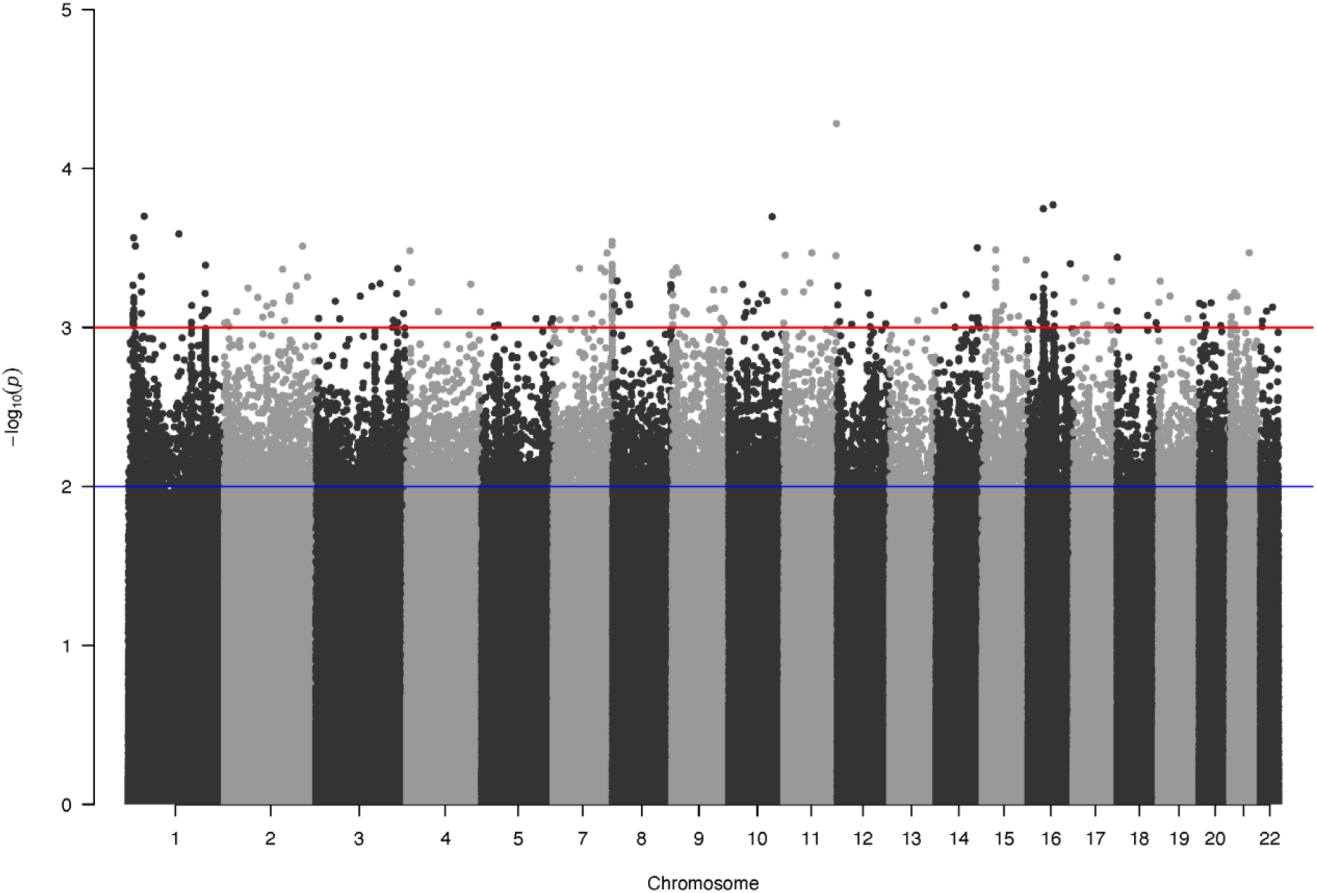
Selection test for SNPs underpinning genetic variance along PC1 of the PCA in Figure 2. The Manhattan plot is derived from a selection scan using PCAngsd of PC1, showing the log‐transformed *p*‐values per chromosome. The blue line indicated a significance line of 0.01 and the red line was 0.001. The analyses are based on the input of genotype likelihoods at 1,093,713 (non‐LD‐pruned) SNPs from the combined Australasian and low‐coverage ecotype datasets.

When identifying and untangling admixture with southwestern Australian killer whales, we ran simple selection tests to see if any admixed regions may be under selection. We used PCANGSD to elucidate the variance that seems to be separating the Australasian killer whales based on genotype likelihoods estimated at 1,093,713 unlinked SNPs. When looking at unpruned SNPs inferred as evolving under selection along PC1, we see several peaks along the genome (Figure 6), however, λ estimates suggest that this analysis only explains 71% of the observed variance (Figure S15) implying there are factors beyond PC1 driving this. Conversely, when identifying ancestral dosage, there were several regions that could be inferred to be evolving under selection. This is somewhat consistent with findings from PCANGSD (Figure 3 and S10). This suggests potential evidence for the selection of admixed regions; however, these loci remain relatively unexplored here.

## 4 Discussion

This study provides direct empirical evidence that sporadic admixture between different genetic backgrounds increases genetic diversity in low‐latitude Australasian killer whale populations. We find that admixture influences present‐day genetic variation within and amongst Australasian killer whale populations. An admixture signal is most strongly evident in the SWA population, and Antarctic‐like ancestry influences patterns of genetic diversity within the population.

Admixture between two populations with distinct genetic backgrounds can maintain higher *N*_e_ (Charlesworth 2009). The genetic variation contributed by the donating population may play a role in the maintenance of individual population health. Specifically, admixture between divergent lineages can provide a genetic ‘rescue’ effect: reducing recessive mutation load (i.e., dominance heterosis) (Fitzpatrick et al. 2020). Longer runs of homozygosity are enriched for homozygous deleterious recessive mutations (Szpiech et al. 2013) and consistent with a genetic rescue effect, we found that recently admixed individuals had fewer long ROH. However, whilst admixture reduces the realised genetic load, it could increase the masked genetic load so that in the event of future inbreeding, introduced deleterious recessive alleles are expressed in homozygous genotypes (Hedrick et al. 2019; Robinson et al. 2019; Szpiech et al. 2019).

Ancestry acts as a source of standing genetic variation and can influence population fitness (Eyre‐Walker and Keightley 2007). Introgressed ancestry can increase adaptive potential (Brauer et al. 2023; Fitzpatrick et al. 2020). We found Antarctic‐like ancestry at higher than expected frequencies in some genomic regions in the SWA population, consistent with, if not conclusive proof of positive selection on Antarctic ancestry. This highlights the need for further research to understand the evolutionary processes shaping the phenotypic and genetic variation within these populations.

Detecting, characterising and untangling admixture between populations remains challenging, especially when the admixing populations are genetically similar (Lawson et al. 2018). Admixture can also become increasingly difficult to identify over time as recombination can erode the length of introgressed tracts (Gopalan et al. 2022). However, recent admixture events can be easier to identify and to characterise their role in shaping the genetic variation of populations by looking for inter‐ancestry patterns (Garcia‐Erill et al. 2023). This is especially true when admixture is between highly distinct genetic backgrounds (e.g., modern and archaic humans, Racimo et al. (2015)).

Signals of recent admixture are evident in SWA genomes and a single NWA genome but are not detected within the tested NZ genomes. This is consistent with coalescent‐based estimates of changes in *N*_e_ (Foote et al. 2021), which combined with other genomic analyses suggest the NZ population to be a small, relatively insular population (Reeves et al. 2022, 2023). In contrast, NWA and SWA populations have relatively stable long‐term *N*_e_ with recent increases (Foote et al. 2021), consistent with potential admixture or population expansion. When assessing admixture for the Australian populations, the reference datasets included lineages genetically similar to those that individuals within the SWA population have admixed with (i.e., Antarctic type C killer whales). Admixture in the genome of NI1 from the NWA population was detected, but the source population was not identified. This is likely due to ancestry being donated from an unsampled lineage not represented within our reference dataset, highlighting the need for increased sampling and sequencing of additional killer whale populations to fully understand the scale of connectivity.

Despite the challenges of identifying ancestry in genomes, we had multiple lines of evidence of sporadic admixture between SWA and Antarctic‐like lineages. Antarctic killer whales have highly divergent genetic backgrounds from other killer whales. Their genomes carry an excess of private (relative to non‐Antarctic killer whales) alleles, some clustered within ‘archaic' tracts (regions of outlier older TMRCA relative to the genome‐wide mean), which contribute to the genetic differentiation between them and other killer whale populations (Foote et al. 2021, 2019). The gene flow from Antarctic‐like lineages represents ~20% of the ancestry of the SWA population, with varying dosages along the genome suggesting ancestral and ongoing gene flow between these lineages.

In summary, this study provides novel insight into the evolutionary history of Australasian killer whales. Analyses of high‐coverage genomes of these low‐latitude killer whales provide evidence that sporadic admixture with distinct genetic lineages is helping to maintain genetic diversity. Thus, admixture could promote genetic rescue within populations of Australasian killer whales. Sporadic gene flow between killer whale populations with distinct genetic backgrounds, as reported here, has been suggested as a broader pattern of behaviour that could explain why populations at low latitudes, low killer whale density and low productivity are able to maintain genetic diversity equal to, or even greater than, in high latitude, high productivity regions with high killer whale density (Foote et al. 2021). Whilst this requires testing (as done here) across more populations to confirm the generality of this pattern amongst killer whale populations, our study does provide support for the concept. More broadly, our study highlights the importance of maintaining connectivity between small populations to their genetic health and persistence.

## Author Contributions

A.D.F. and I.M.R. conceived the study. J.A.T., K.A.S. and E.L.B. provided samples from the contemporary Australasian populations. I.M.R. conducted laboratory work. I.M.R. conducted genomic and bioinformatic analysis with guidance from A.D.F. and J.S.‐C., I.M.R. wrote the manuscript with input from all coauthors.

## Conflicts of Interest

The authors declare no conflicts of interest.

## Supporting information


Figure S1‐S15.

Table S1‐S11.

## Data Availability

Sequencing data generated from Australian samples for this study has been archived at the National Centre for Biotechnology Information (www.ncbi.nlm.nih.gov) under the Orcanomics BioProject (NCBI accession: PRJNA531206). Sequencing data generated from New Zealand samples have been uploaded to the Genomics Aotearoa Repository (https://www.genomics‐aotearoa.org.nz: accession: https://doi.org/10.57748/3vba‐rg35).
