# Supplementary material for: Admixture Increases Genetic Diversity and Adaptive Potential in Australasian Killer Whales
Source: Mol Ecol. 2025 Feb 28;34(23):e17689. doi: 10.1111/mec.17689 (PMC12684329; doi:10.1111/mec.17689)
Supplement: Supplementary file 1 — Figure S1‐S15. [file MEC-34-e17689-s001.pdf]

# Supplementary Material

# Admixture increases genetic diversity and adaptive potential in Australasian killer whales

Isabella M. Reeves[1,2*], John A. Totterdell[2], Jonathan Sandoval-Castillo[3], Emma L. Betty[4], Karen A. Stockin[4], Ramari Oliphant Stewart[5], Muriel Johnstone[6] and Andrew D. Foote[7*]

[1]Flinders University, College of Science and Engineering, Bedford Park 5042 South Australia

[2]Cetacean Research Centre (CETREC WA), PO Box 1029 Esperance, 6450 Western Australia

[3]Molecular Ecology Laboratory, College of Science and Engineering, Flinders University, Bedford Park, SA 5042, Australia

[4]Cetacean Ecology Research Group, College of Sciences, Massey University, Auckland, New Zealand

[5] Te Kauika Tangaroa Charitable Trust, Westland, New Zealand

[6]Ōraka-Aparima Rūnaka, Aparima (Riverton), New Zealand

[7]Centre for Ecological and Evolutionary Synthesis, Department of Biosciences, University of Oslo, 0316 Oslo, Norway

***Corresponding authors**

Isabella Reeves

College of Science and Engineering, Flinders University, Bedford Park, South Australia 5042

Email: isabella.reeves@flinders.edu.au

Andrew Foote

Centre for Ecological and Evolutionary Synthesis, Department of Biosciences, University of Oslo, 0316 Oslo, Norway

Email: andrew.foote@ibv.uio.no

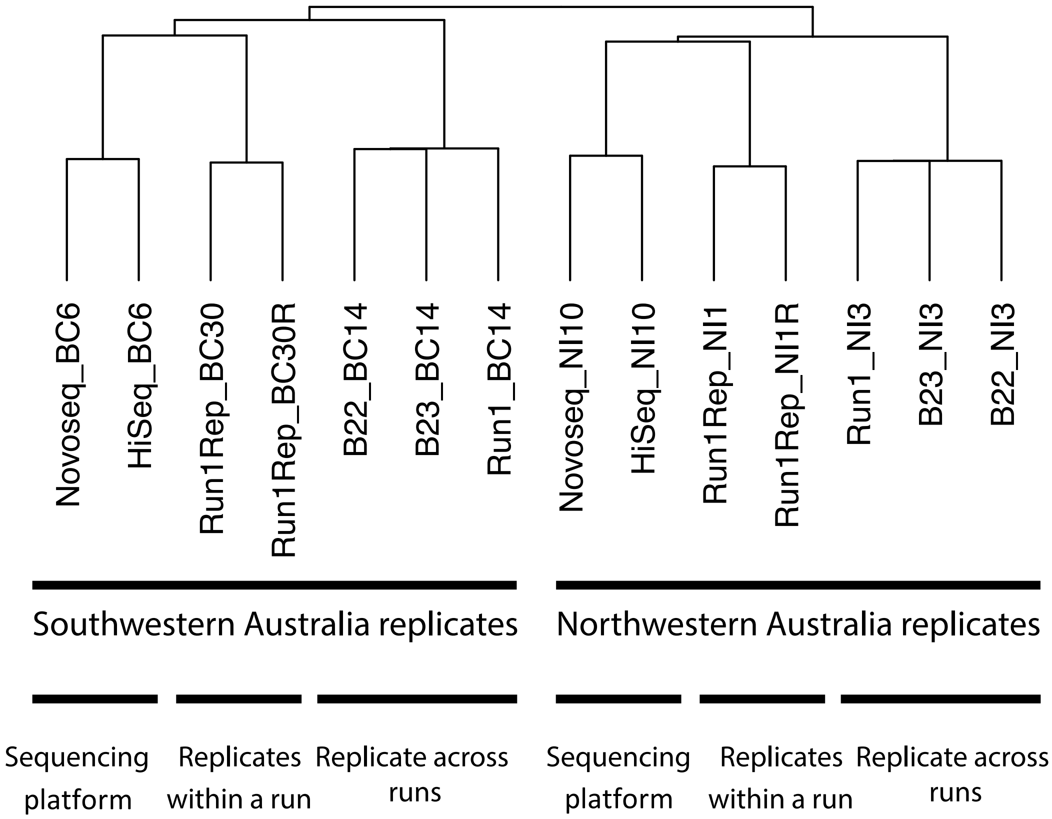


**Figure S1: Assessing batch effects.**

Neighbour-joining tree constructed from covariance estimates, in which a random allele is selected from each polymorphic site of each genome. The approach results in variation even when sampling from the same individual but should approximately reconstruct relative covariance among samples. Branch lengths of the NJ tree are not proportional to covariance, but rather illustrate branching order. The replicates include those on the same sequencing run, and on different sequencing runs of an Illumina HiSeq 4000 platform; and those across different runs on the Illumina NovaSeq 6000 and HiSeq 4000 platforms in different labs, as part of different studies (Foote et al. 2021; Reeves et al. 2023). The clustering of runs by individual rather than runs provides confidence that any batch effects are expected to be minor. 'Run 1' indicates sample was sequenced in 2021 at Novogene (Singapore), B22 indicated sample was sequenced in a different batch in 2022 at QB3 Berkley and B23 indicates that samples were sequenced again in 2023 at QB3 Berkley. Run 1' indicates samples sequenced in 2021 at Novogene (Singapore), while 'B22' and 'B23' refer to samples sequenced at QB3 Berkeley in 2022 and 2023, respectively.

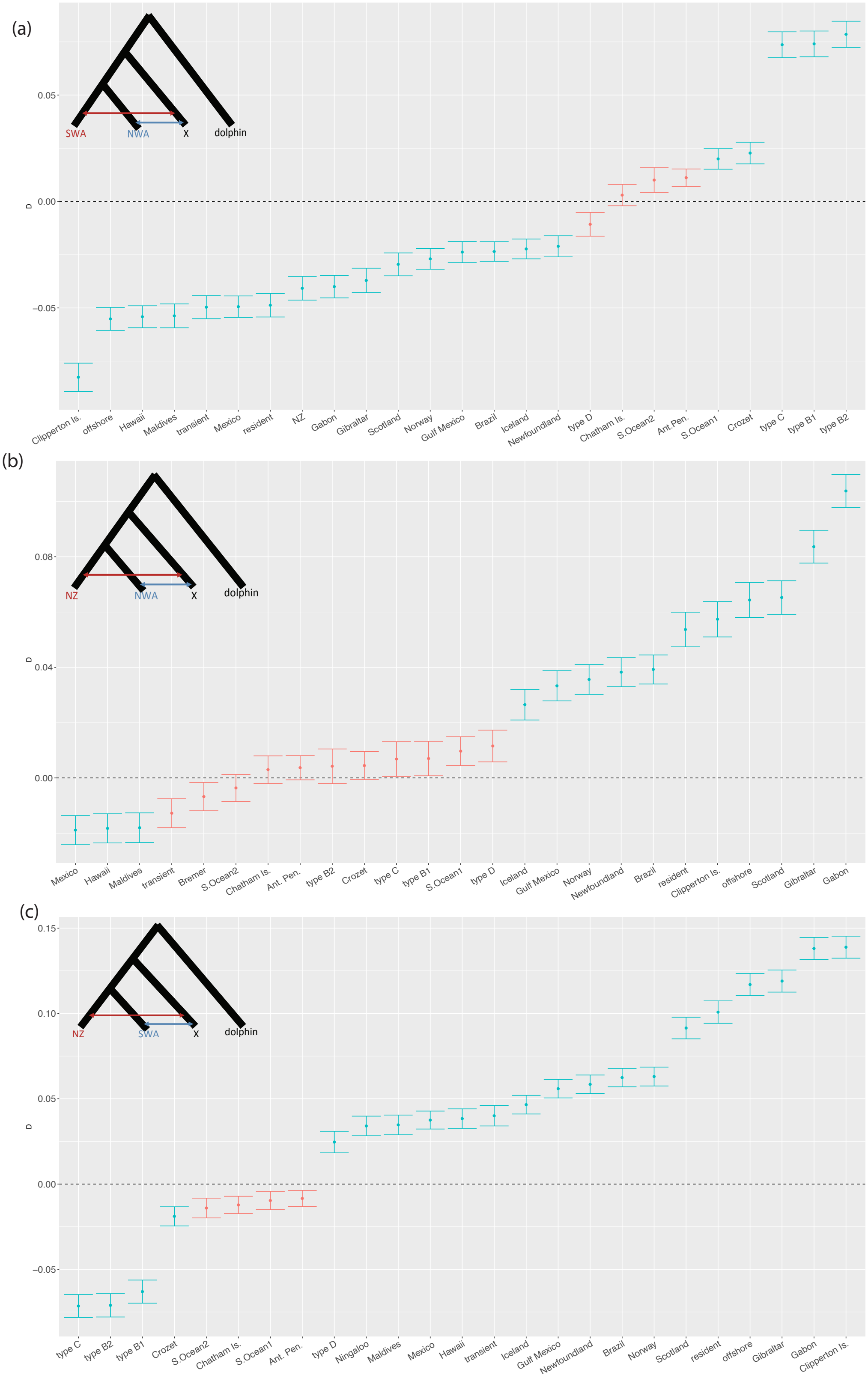


**Figure S2**: *D*-statistic forms tested with a single sample per location, with Z-scores-3 < Z > 3, being significant (blue) and non-significant (red). **(a)** *D*(SWA, NWA, X, dolphin) with 21 significant tests, **(b)** *D*(NZ, NWA, X, dolphin) with 21 significant tests and **(c)** *D*(NZ, SWA, X, dolphin) with 14 significant tests. 'SWA', is Southwestern Australia, 'NWA' is Northwestern Australia and 'NZ' New Zealand.

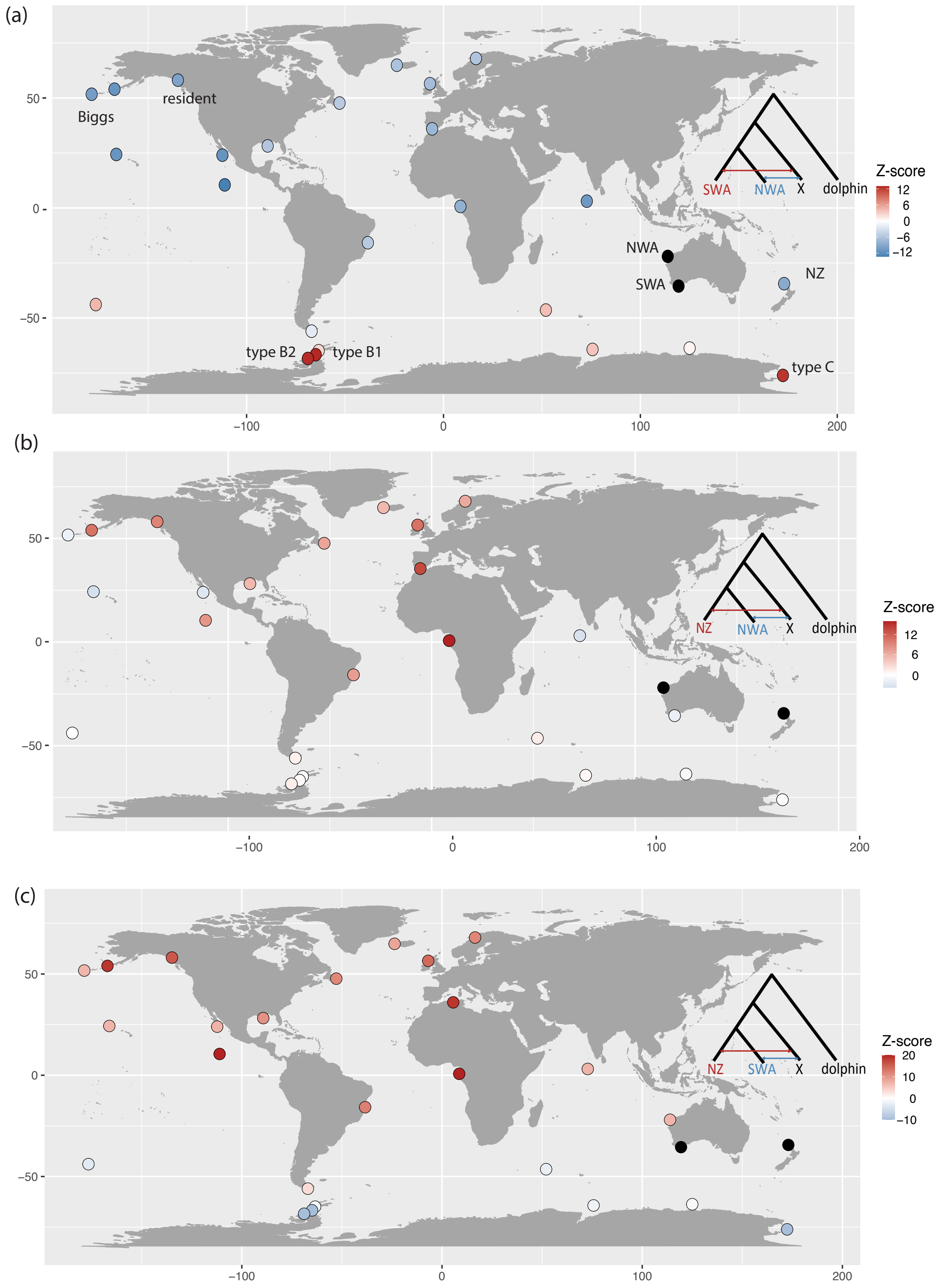


**Figure S3:** A comparison of *D*-statistic form **(a)** *D*(SWA, NWA, X, dolphin), **(b)** *D*(NZ, NWA, X dolphin) and **(c)** *D*(NZ, SWA, X dolphin) for Australasian killer whales compared to a global reference dataset. Statistical significance is indicated by a Z-score >3 or <-3. These *D*-statistic estimates are based off data mapped the alternative chromosomal reference genome assembly of a Norwegian killer whale (Foote et al. 2022 doi: 10.12688/ wellcomeopenres.18278.1) to examine effects of reference bias. 'SWA' is Southwestern Australia, 'NWA' is Northwestern Australia and 'NZ' is New Zealand. Dots are representative of sample locality.

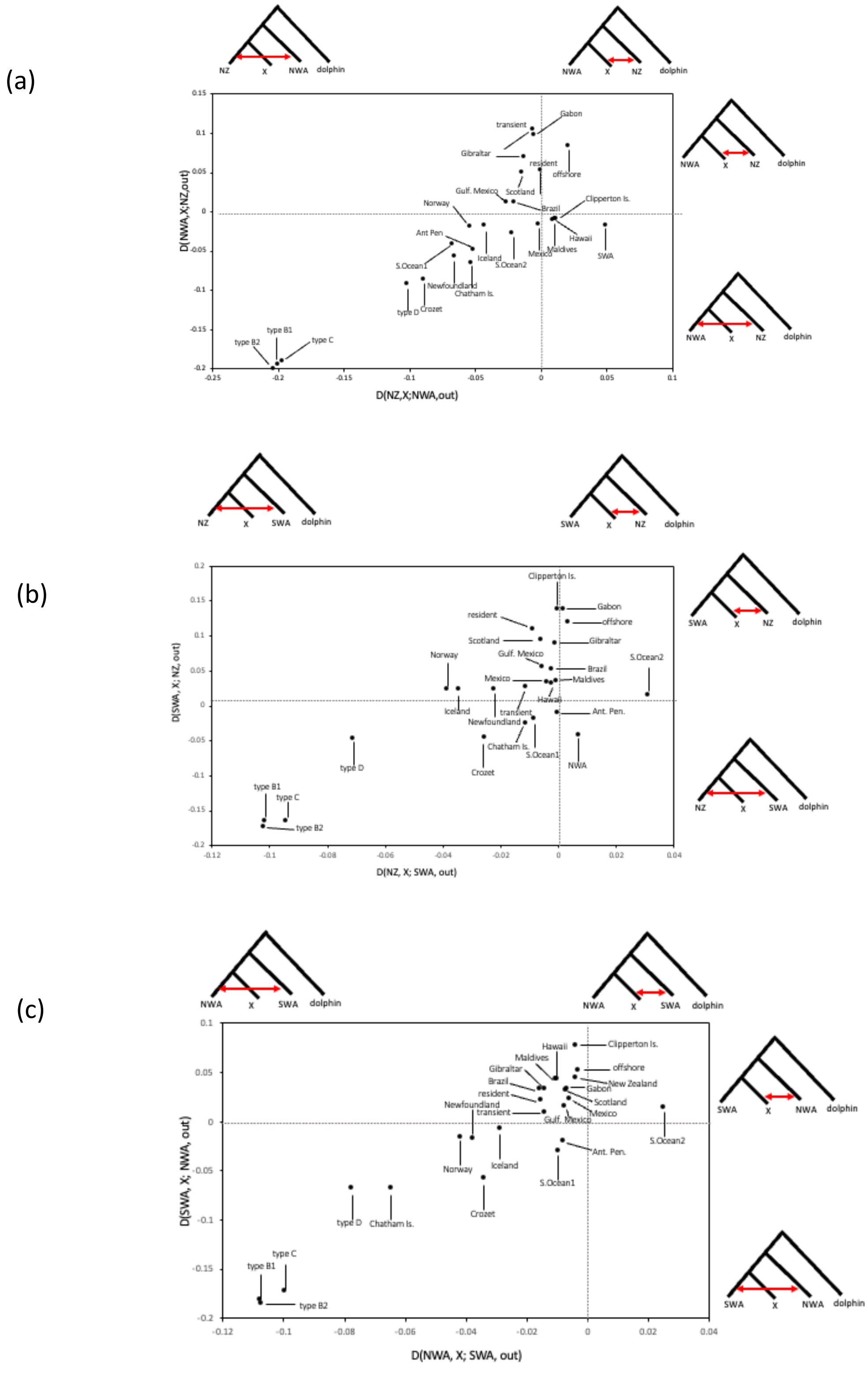


**Figure S4:** A comparison of *D*-statistic form *D*(pop1, X, pop2 dolphin) and *D*(pop1 X, pop2, dolphin).Where pop1 or 2 represents a single Australasian sample from one of the three populations. Positive values along the x-axis indicate X shared an excess of derived alleles with the pop2. Negative *D*-statistics indicate that pop1 and pop2 shared an excess of derived alleles than with X. Please note ‘transient’ whales here are referred to as ‘Biggs’ killer whales elsewhere in this manuscript.

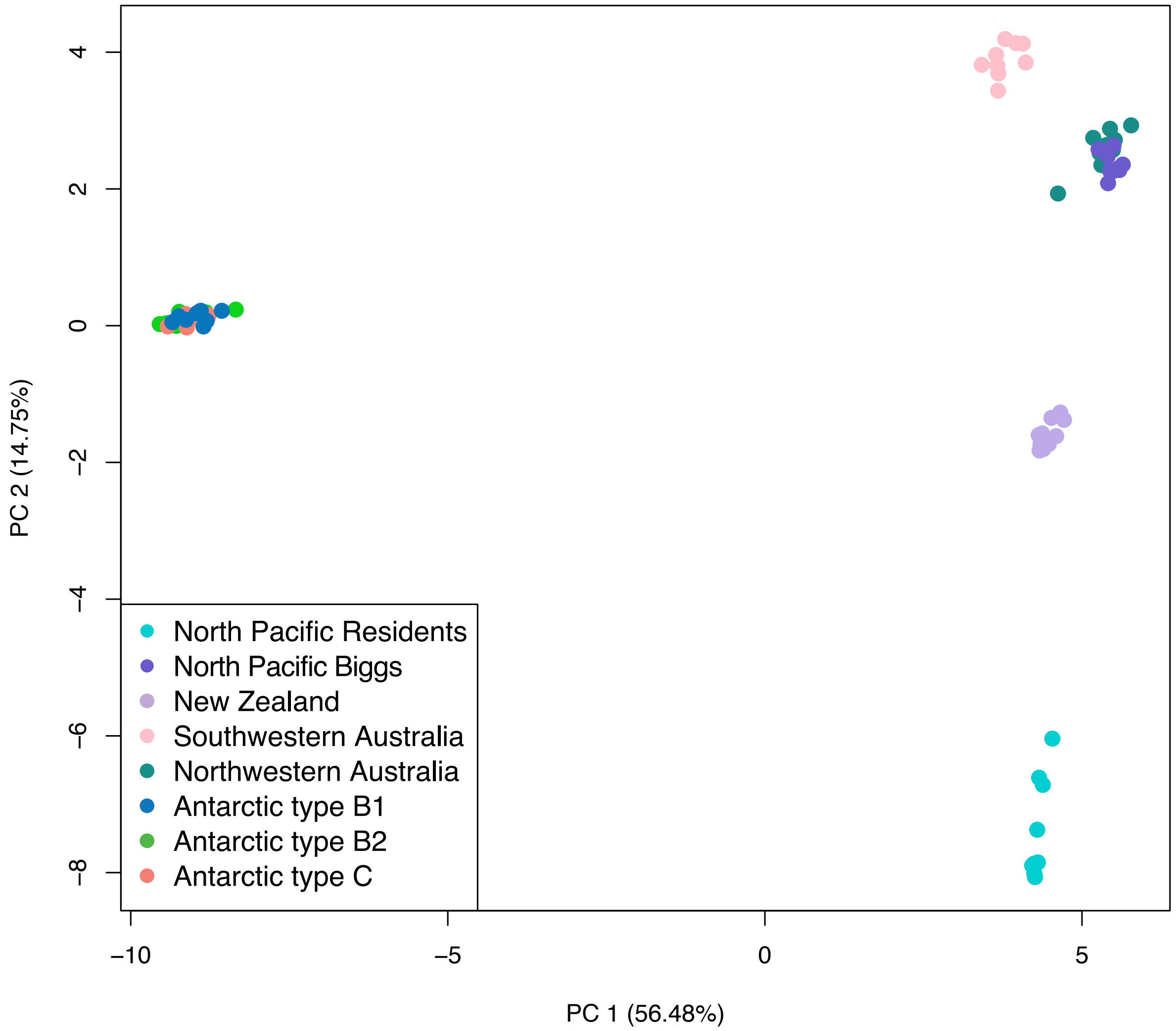


**Figure S5:** PCA plot of all population-level whole genome data available for killer whales based on 18,355 SNPs. As consistent with previous studies Antarctic types drive the major axis of differentiation within killer whales along PC1 (56.48%), followed by the residents along PC2 (14.75%).

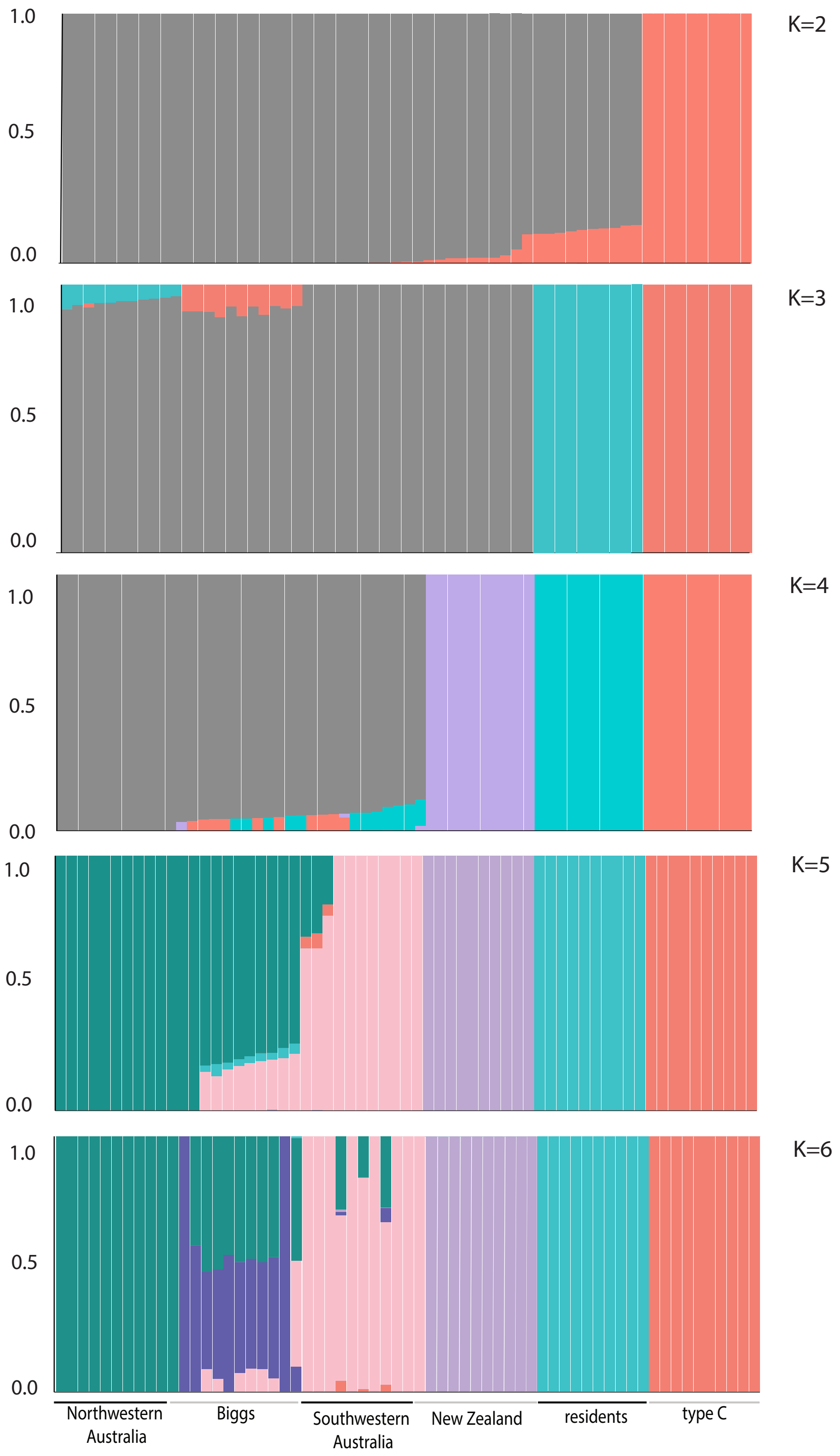


**Figure S6:** Population structure assignment based on 18,355 putatively neutral SNPs for killer whales from North Pacific residents, North Pacific Biggs, Antarctic type C, New Zealand, southwestern and northwestern Australia. Plots depict K=2-6.

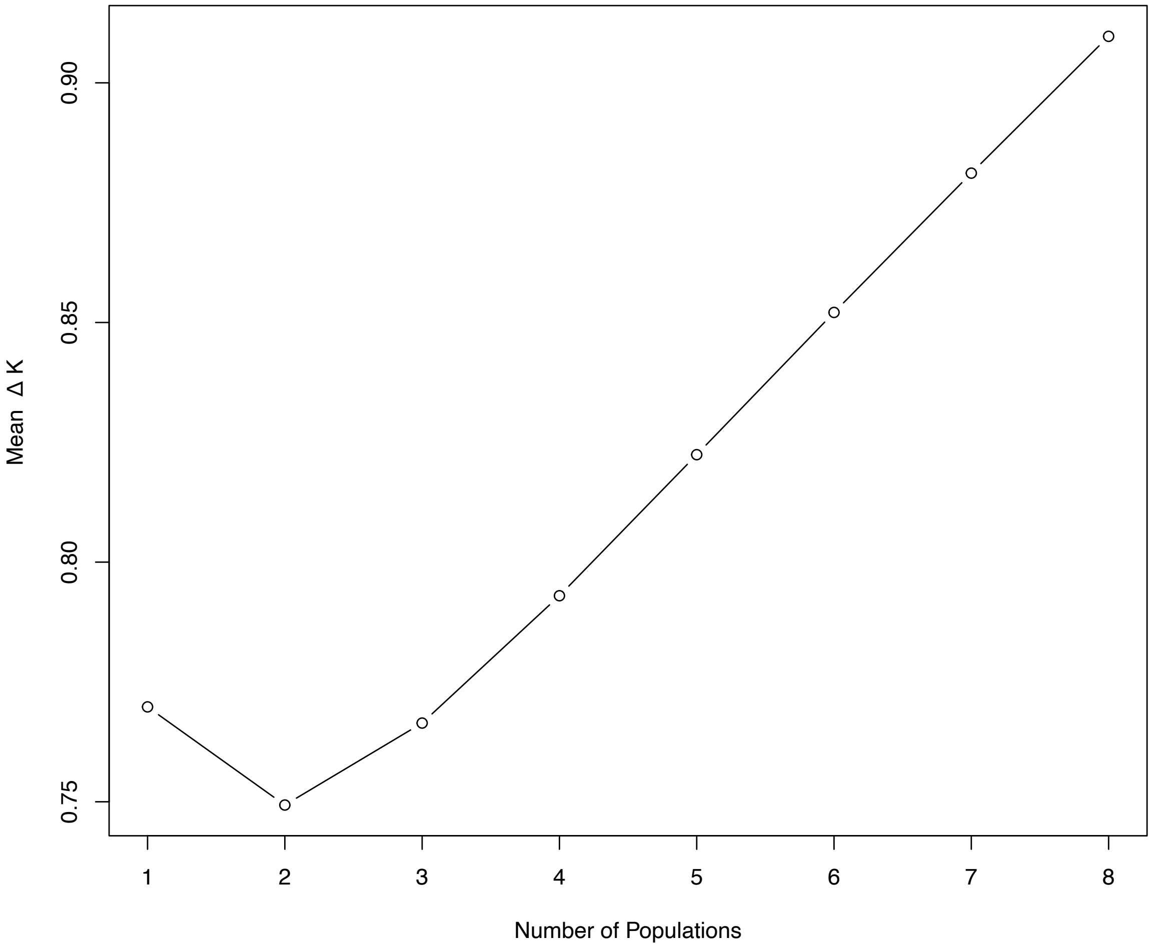


**Figure S7:** Mean Delta *K* to identify the best support number of clusters of admixture results based on Figure S6 NGSadmix output.

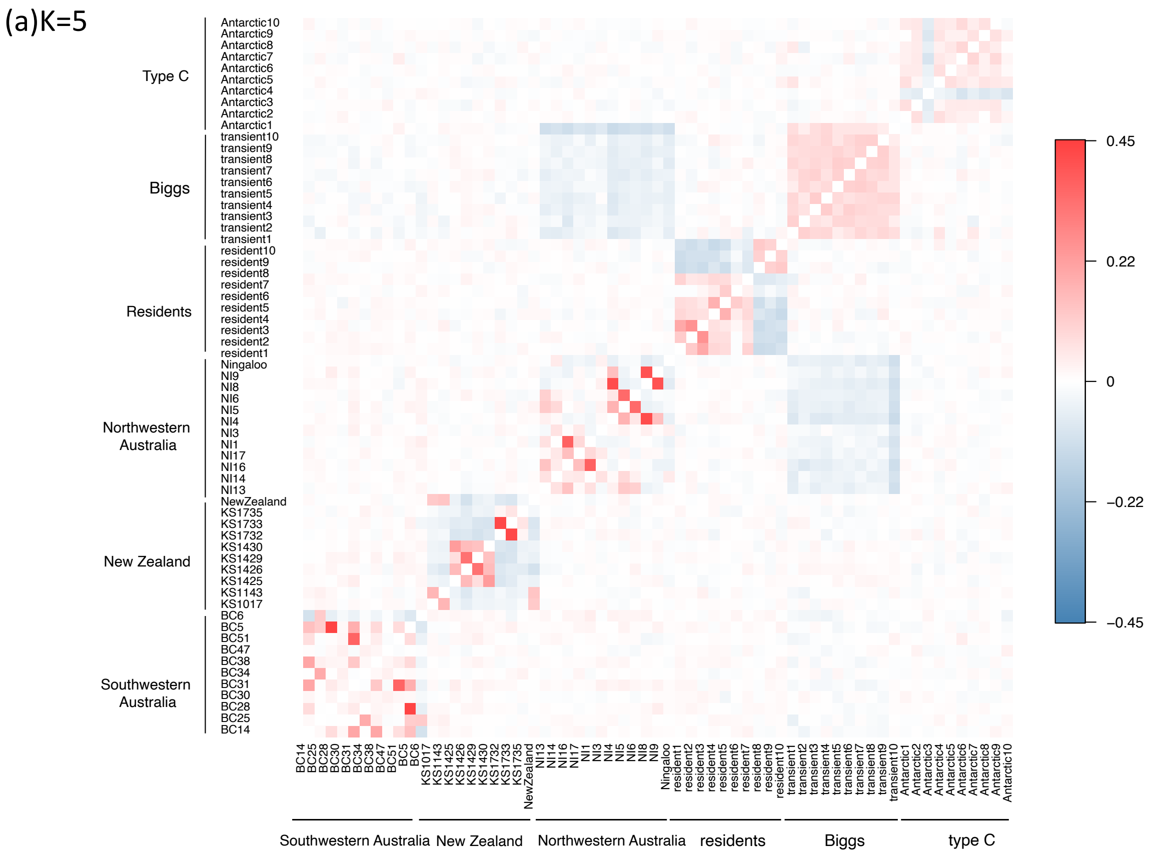
(a)K=5
Type C
Biggs
Residents
Northwestern Australia
New Zealand
Southwestern Australia
Southwestern Australia
New Zealand
Northwestern Australia
residents
Biggs
type C
0.45
0.22
0
−0.22
−0.45

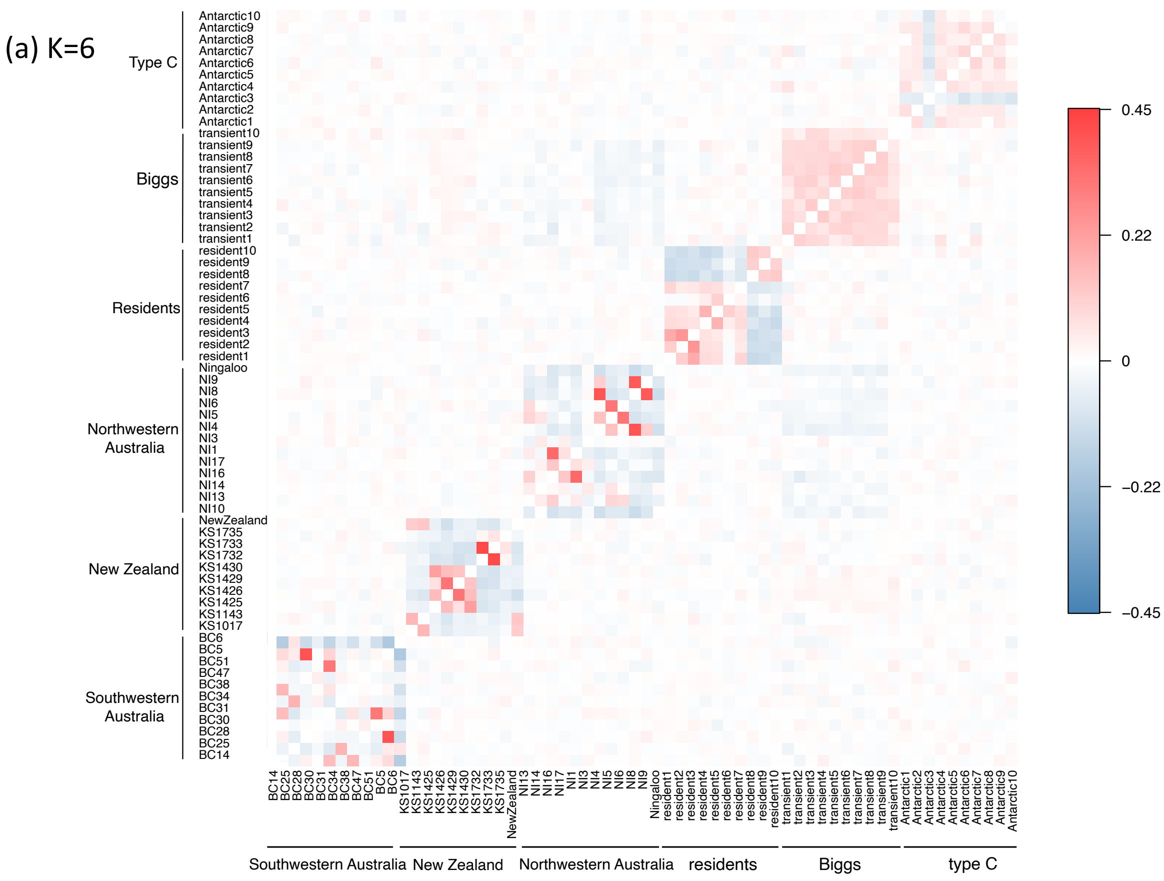
(a) K=6
Type C
Biggs
Residents
Northwestern Australia
New Zealand
Southwestern Australia
Southwestern Australia
New Zealand
Northwestern Australia
residents
Biggs
type C
0.45
0.22
0
−0.22
−0.45

**Figure S8:** Evaluation of model fit of the admixture results using evalAdmix based on 18,355 putatively neutral SNPs for both (a) K=5 and (b) K=6 as the correlation of residuals. Positive correlation values (red) and negative (blue) indicate poor fits to the inferred admixture proportions based on Figure 5S NGSadmix outputs, whereas 0 or close indicate good fit to admixture model.

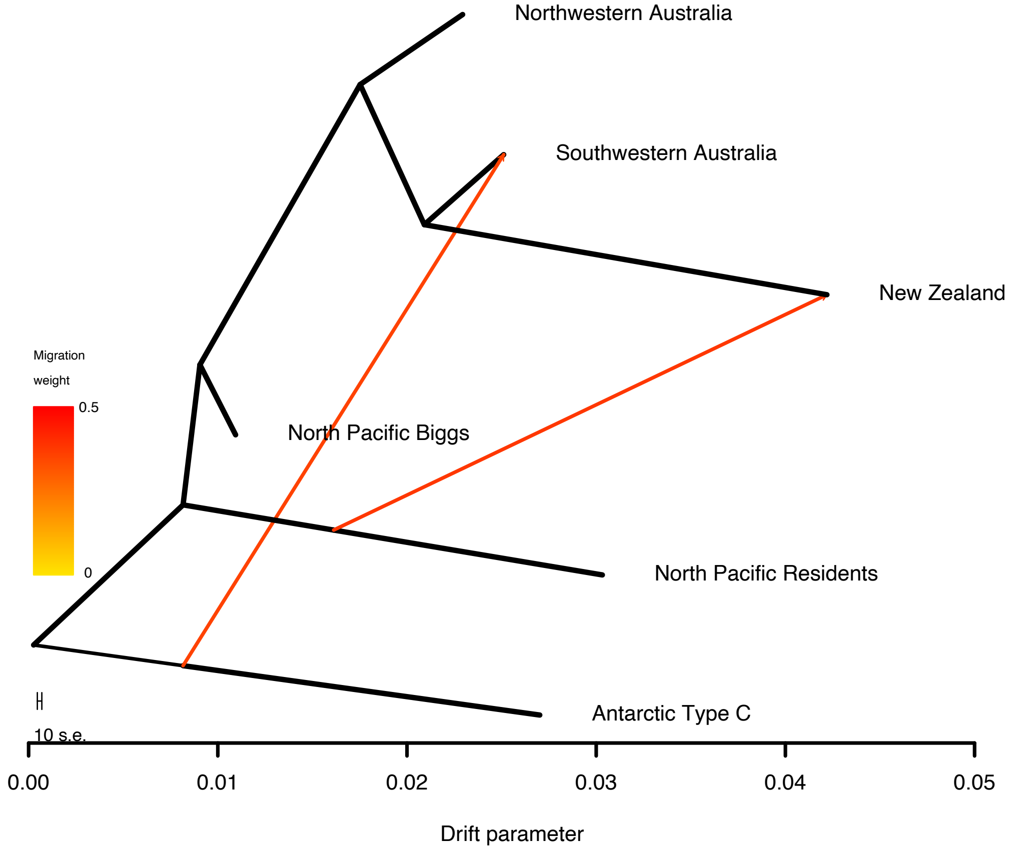


**Figure S9:** Bifurcating maximum likelihood tree with Gaussian approximation for Australasian and reference population whole genome datasets based on 1,000,517 SNPs using 1,000 SNP blocks.

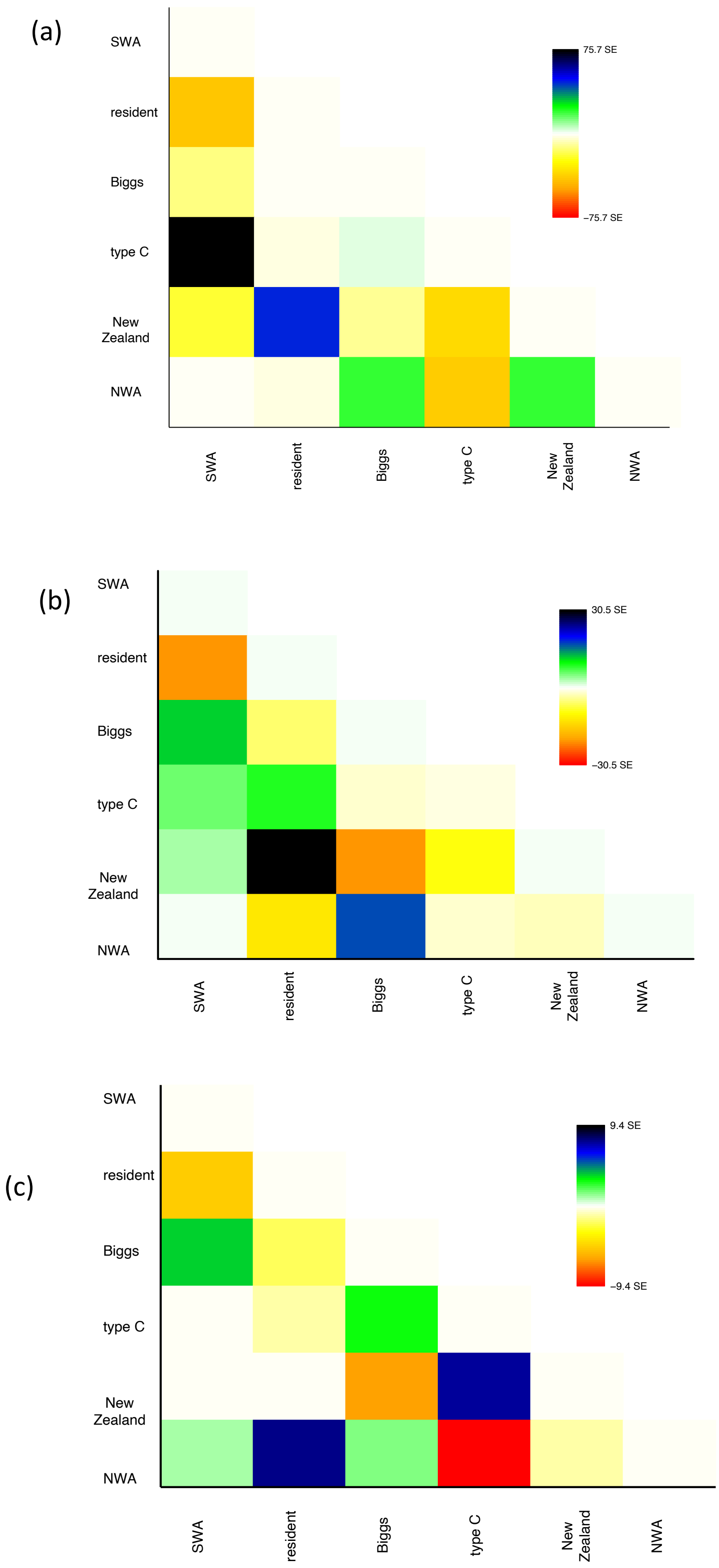


**Figure S10**: Residual fit of TreeMix bifurcating trees Figure 1 and S9, based on Australasian and reference population killer whale whole genome datasets based on 1,000,517 SNPs using 1,000 SNP blocks for **(a)** no migration edges, **(b)** one migration edge, and **(c)** two migration edges. ‘SWA’ is Southwestern Australia, ‘NWA’ is Northwestern Australia and ‘NZ’ is New Zealand.

(a)

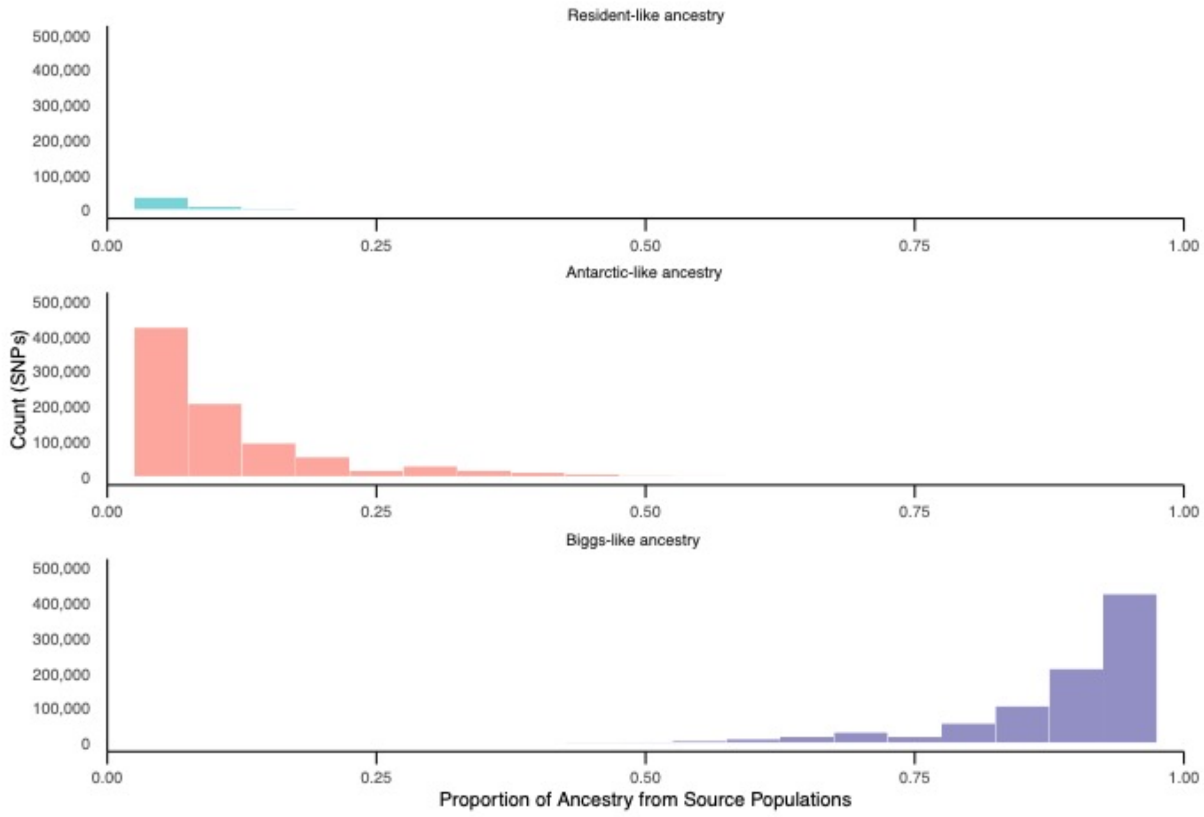


(b)

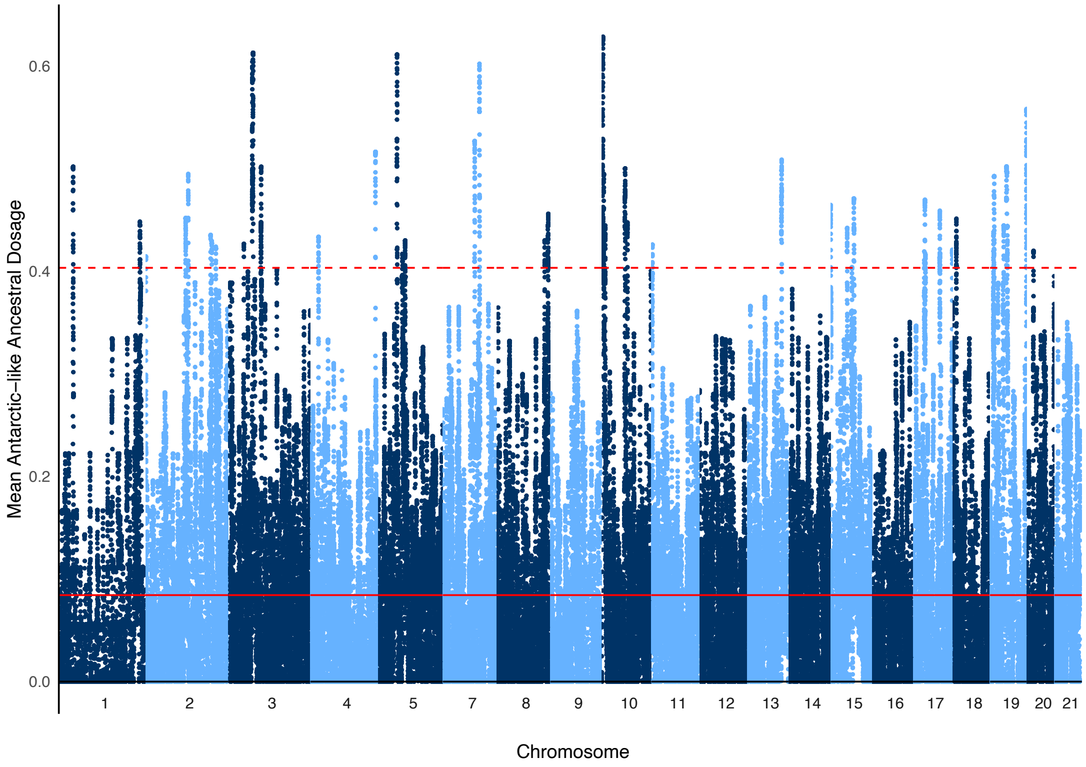


**Figure S11**: The distribution of southwestern Australian killer whale local ancestry across the genome by SNP (n=1,000,517) by reference population for **(a)** SNP dosage genome-wide and **(b)** Mean Antarctic-like ancestry across the genome per chromosome for 10 generations. The solid red line is the mean ancestry proportion, and the dotted lines are ± 3 standard deviations based on 50KB sliding windows and 10KB stepping size.

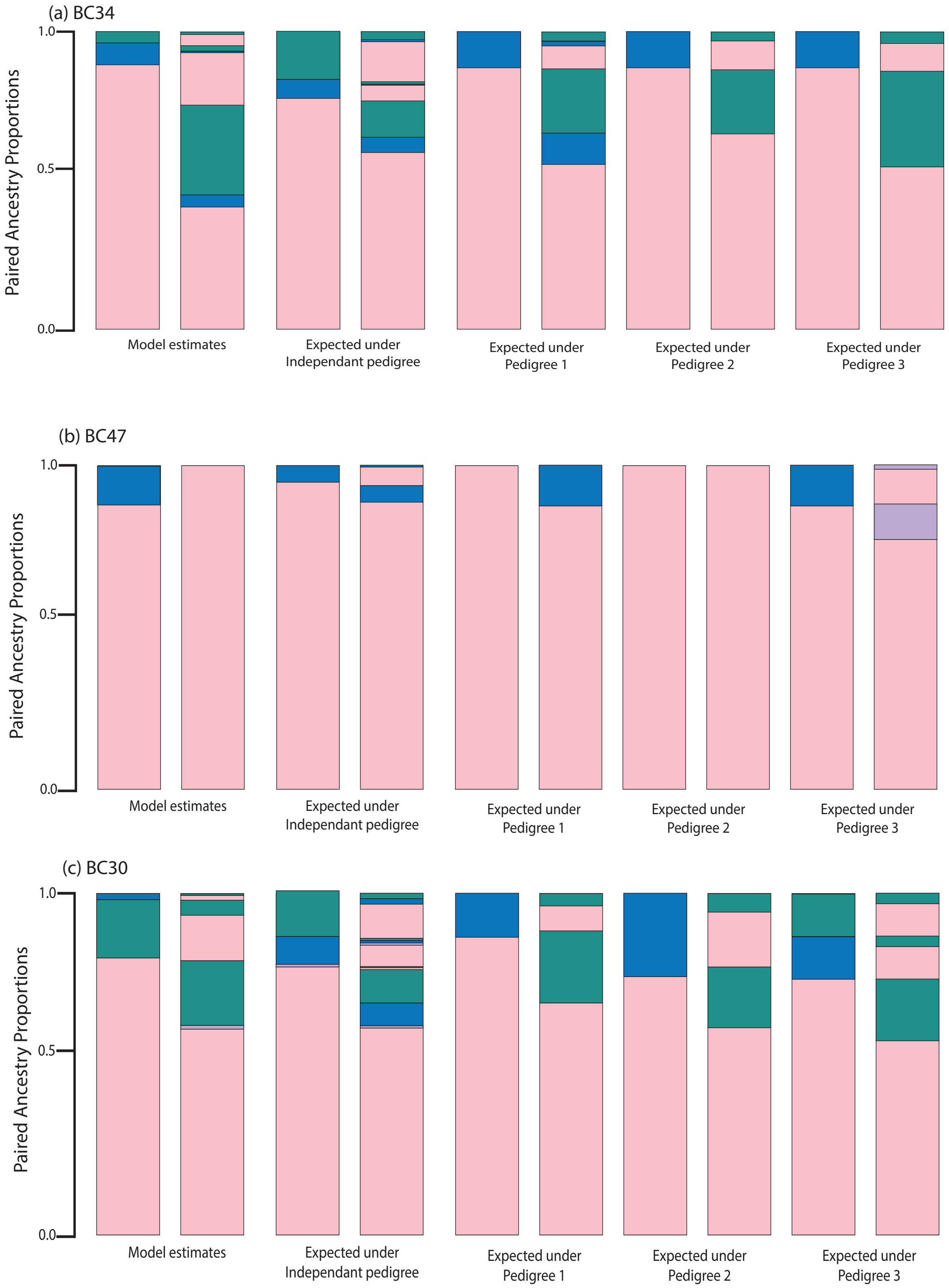


**Figure S12:** Paired ancestry proportions model estimates based on estimates of admixture of recently admixed southwestern Australian killer whales assuming K=7 ancestral components based on 18,355 unlinked SNPs. **(a)** is BC34, **(b)** is BC47, and **(c)** BC30. Note that pink is southwestern Australian ancestry, blue is Antarctic type B-like ancestry, green is northwestern Australian ancestry, and purple is New Zealand ancestry.

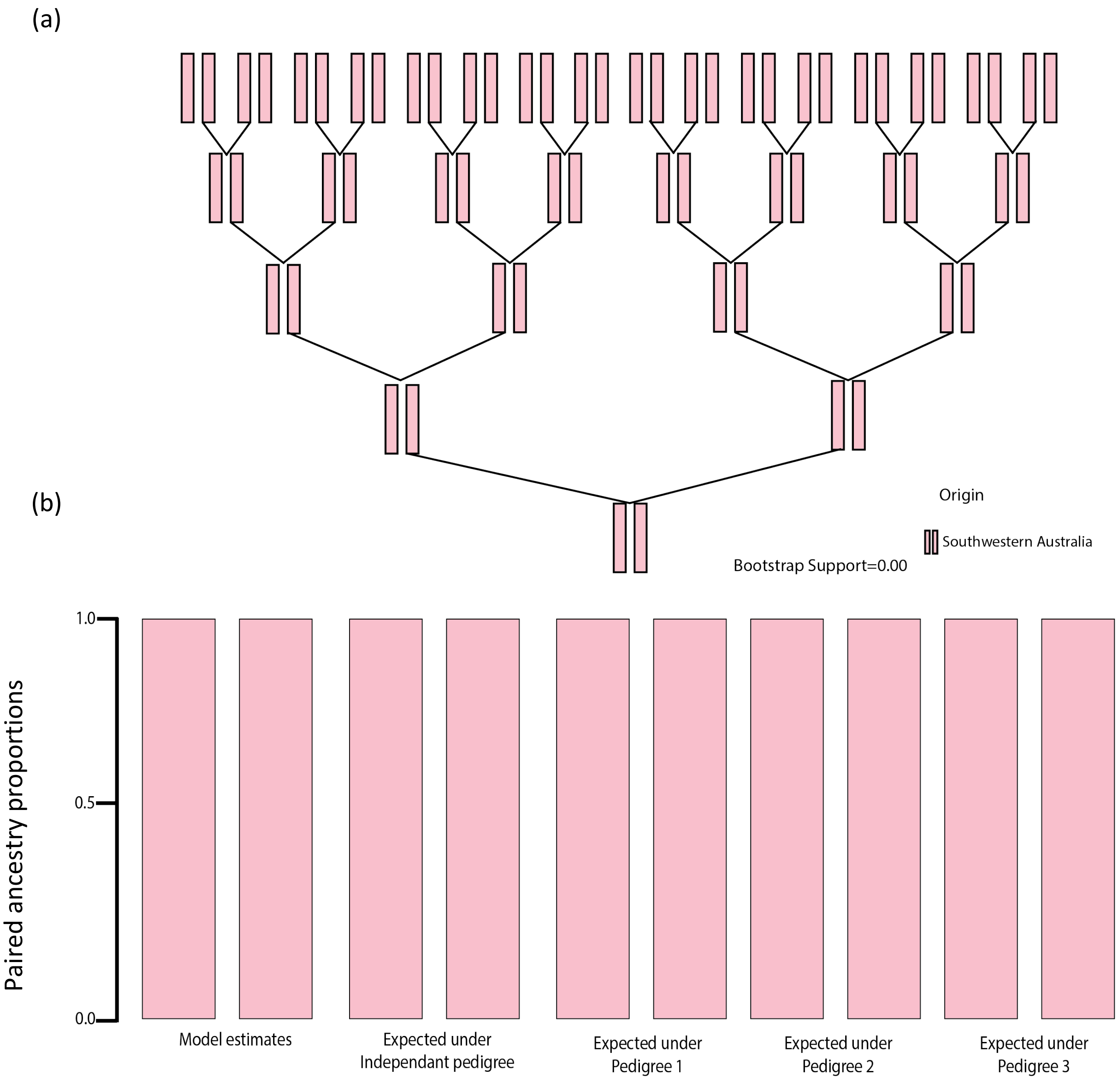


**Figure S13:** Estimates of admixture of not recently admixed southwestern Australia killer whales assuming K=7 ancestral components. **(a)** Most compatible recent admixture pedigrees including their bootstrap support based on 18,355 unlinked SNPs and **(b)** Paired ancestry model estimates with expected proportion under an independent pedigree and the pedigrees estimated by apoh. This was repeated for candidates not recently admixed genomes (B14, BC28, and BC51) and these analyses were identical between the three of the individual genomes therefore only this figure is shown as representative.

(a)

(b)

(c)

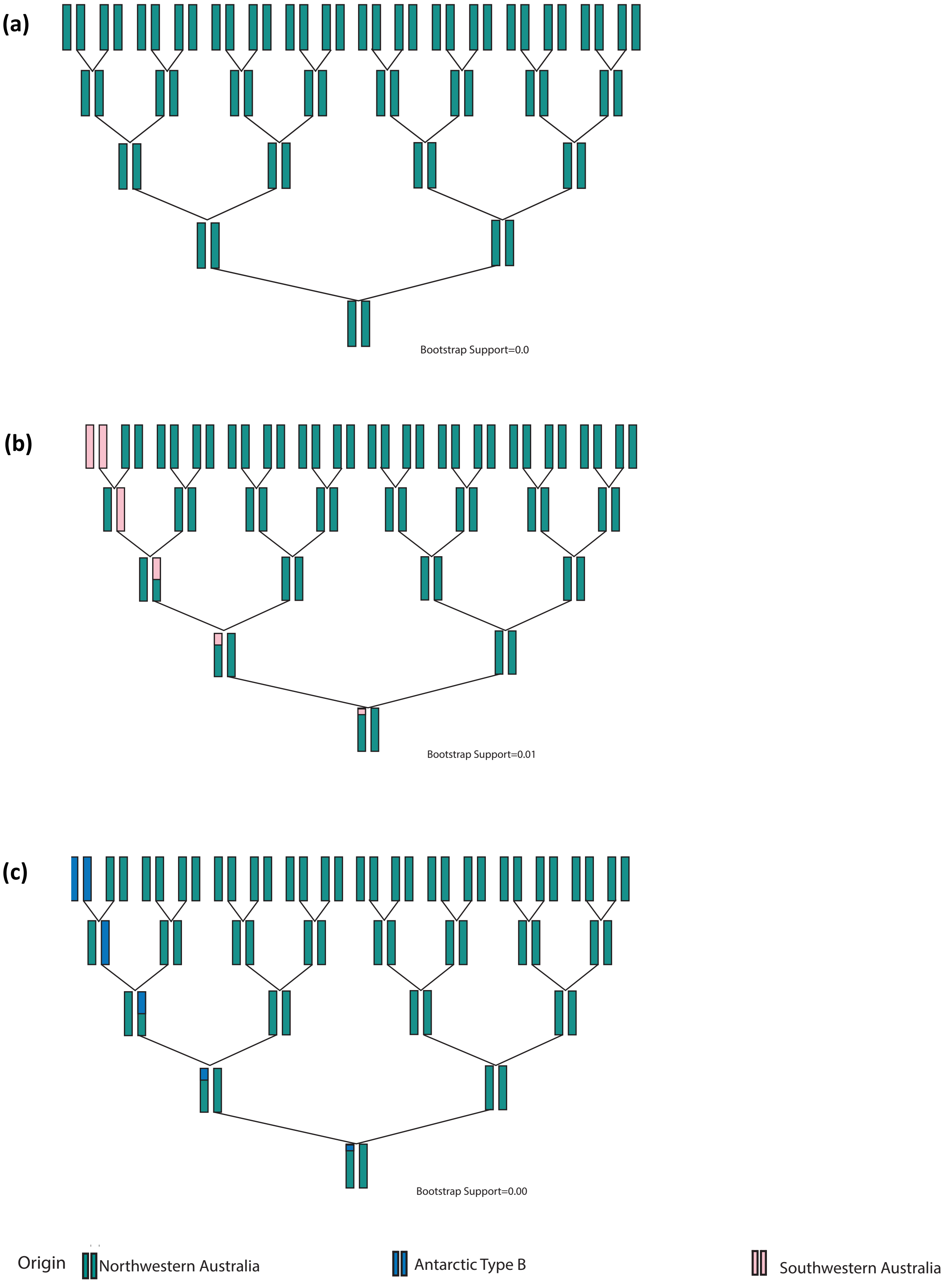


**Figure S14:** Estimates of recent admixture of NI1 from northwestern Australia assuming K=7 ancestral components. (a-c) Most compatible recent admixture pedigrees including their bootstrap support value based on 18,355 unlinked SNPs. Table S8 suggests no models here fit the data well, likely due to

not having representative samples from lineages they have admixed with. Note that pink is southwestern, Australian ancestry blue is type B ancestry and green is northwestern Australian ancestry.

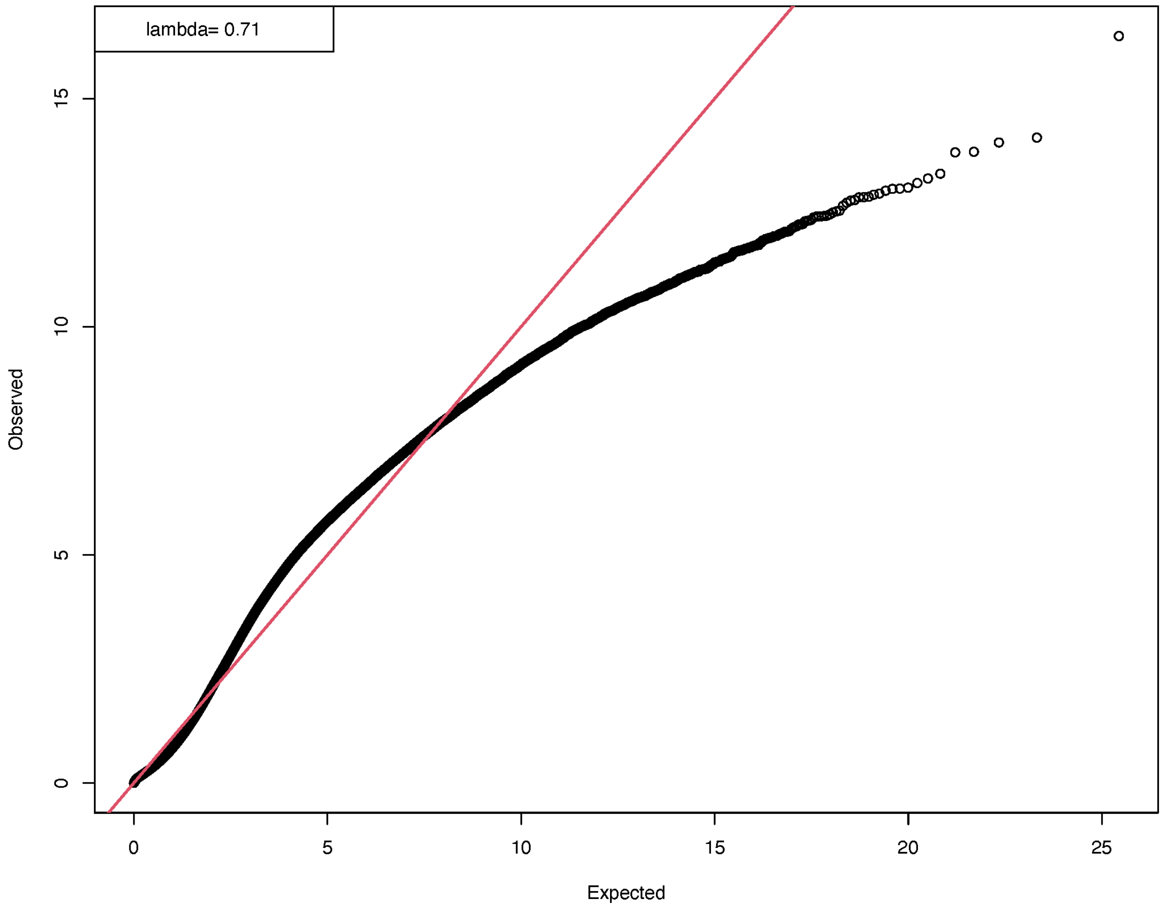


**Figure S15:** Observed vs expected fit of data based on PC1 selection scan SNPs based on -log10 transformed p-values on Australasian dataset and reference data, using PCAngsd -selection function based on 1,093,713 SNPs based on genotype likelihoods.
